# Analysis of Argonaute 4-Associated Long Non-Coding RNA in *Arabidopsis thaliana* Sheds Novel Insights into Gene Regulation through RNA-Directed DNA Methylation

**DOI:** 10.3390/genes8080198

**Published:** 2017-08-07

**Authors:** Phil Chi Khang Au, Elizabeth S. Dennis, Ming-Bo Wang

**Affiliations:** Commonwealth Scientific and Industrial Research Organisation Agriculture, Canberra, Australian Capital Territory 2601, Australia; Phil.au@outlook.com (P.C.K.A.); liz.dennis@csiro.au (E.S.D.)

**Keywords:** RNA-directed DNA methylation, AGO4, noncoding RNA, plants

## Abstract

RNA-directed DNA methylation (RdDM) is a plant-specific de novo DNA methylation mechanism that requires long noncoding RNA (lncRNA) as scaffold to define target genomic loci. While the role of RdDM in maintaining genome stability is well established, how it regulates protein-coding genes remains poorly understood and few RdDM target genes have been identified. In this study, we obtained sequences of RdDM-associated lncRNAs using nuclear RNA immunoprecipitation against ARGONAUTE 4 (AGO4), a key component of RdDM that binds specifically with the lncRNA. Comparison of these lncRNAs with gene expression data of RdDM mutants identified novel RdDM target genes. Surprisingly, a large proportion of these target genes were repressed in RdDM mutants suggesting that they are normally activated by RdDM. These RdDM-activated genes are more enriched for gene body lncRNA than the RdDM-repressed genes. Histone modification and RNA analyses of several RdDM-activated stress response genes detected increased levels of active histone mark and short RNA transcript in the lncRNA-overlapping gene body regions in the *ago4* mutant despite the repressed expression of these genes. These results suggest that RdDM, or AGO4, may play a role in maintaining or activating stress response gene expression by directing gene body chromatin modification preventing cryptic transcription.

## 1. Introduction

RNA-directed DNA methylation (RdDM) can result in transcriptional gene silencing in plants [1,2,3]. Of the three major RNA silencing pathways in plants, RdDM is the sole pathway capable of mediating DNA methylation [4,5,6,7,8]. RdDM plays a fundamental role in plant defense against invasive DNA and in maintaining genome stability by silencing transposons and repetitive sequences. RdDM has also been shown to regulate protein-coding genes [9,10], but this aspect of RdDM function has not been well studied, and very few RdDM-targeted genes have been identified. Much effort over the past decade has been attributed to the characterization of the components and the molecular mechanism of RdDM allowing a canonical pathway to be well established. RdDM is induced by 24-nt small interfering RNAs (siRNAs), generated by the combined function of the plant-specific RNA POLYMERASE IV (POL IV) [4,5,7], RNA-DEPENDENT RNA POLYMERASE 2 (RDR2) [8,11] and DICER-LIKE 3 (DCL3) [11,12]. POL IV transcribes single stranded RNA, which becomes converted into double-stranded RNA (dsRNA) by RDR2. DCL3, the main RNase III endoribonuclease of RdDM, cleaves these dsRNA into 24 nt siRNAs. These 24-nt siRNAs are loaded onto ARGONAUTE 4 (AGO4) to form an effector complex also known as RNA-induced silencing complex (RISC) [13,14,15], which is recruited to genomic loci through physical interaction with longer non-coding RNAs (lncRNAs) that are transcribed by another plant-specific RNA POLYMERASE V (POL V) [16,17]. These lncRNAs function as a scaffold to determine the exact target genomic region at which a DNA methylation enzyme, DOMAINS REARRANGED METHYLTRANSFERASE 2 (DRM2), catalyzes cytosine methylation at all sequence contexts (CG, CHG and CHH, where H represents A, C or T). DNA methylation at these loci maintains transcription in a repressive state and therefore plays a fundamental role in fine-tuning the expression of genomic elements. Genome-wide methylation analyses indicate that RdDM accounts for 30% of all DNA methylation in *Arabidopsis* [18].

RdDM mutants of *Arabidopsis* generally do not show phenotypic abnormalities and develop like their wild-type (WT) counterparts; however, recent studies using plants carrying mutations in genes required for functional RdDM have implicated this pathway in the regulation of genes involved in conferring plant immunity. *Arabidopsis* plants deficient in AGO4 are highly susceptible to bacterial (*Pseudomonas syringae*) infection [19]. Mutants defective in RNA POL V and RDR2 are susceptible to the fungi *Botrytis cinerea* and *Plectosphaerella cucumerina* [20]. While it is widely accepted that plants defend against invasive pathogens through triggering complex signaling cascades regulated by salicylic acid, jasmonic acid and ethylene which regulate the expression level of defense-related genes [21,22], these recent studies clearly demonstrate that RdDM mutants are more susceptible to infection and highlight an alternate pathway that determines plant immunity. How RdDM, known to repress genes, is involved in plant immunity remains unclear.

While previous genome-wide profiling and AGO4 pull-down analyses have identified large populations of 24-nt siRNAs, the scaffold lncRNAs have not been well characterized. This has hindered the identification of RdDM-regulated genes in plants, particularly protein-coding genes. Here, using nuclear RNA-immunoprecipitation, Illumina deep sequencing and bioinformatics analyses, along with RT-PCR analysis, we established a high-quality library of AGO4-associated long ncRNAs, and identified novel RdDM regulated protein-coding genes and those involved in disease response. We also generated evidence implicating the RdDM pathway in the activation of protein-coding genes through gene body methylation or chromatin modification, particularly those involved in stress response.

## 2. Materials and Methods

### 2.1. Plant Material and Growth Conditions

All wild-type, transgenic and mutant *Arabidopsis thaliana* plants were grown in controlled conditions under 16 h light at 22 °C on MS medium/agar. The *dcl* and *rdr* mutants were described previously in Wang et al. [23]; the *rdd* (Col-0 background with *ros1*, *dml2*, *dml3*; SALK_045303, SALK_131712, SALK_056440, respectively), *nrpd1* (*nrpd1-3*), *nrpe1* (*drd3-7*) and *ago4-2* (*ocp11*) mutants were kindly provided by Jian-Kang Zhu, David Baulcombe, Majori Matzke, and Pablo Vera, respectively. Aerial tissues of three-week-old seedlings were collected for RNA and protein expression analyses.

### 2.2. RNA Extraction and cDNA Synthesis

*Arabidopsis* tissues were ground into fine powder under liquid nitrogen using a mortar and pestle. Total RNA was extracted using TRIzol (Ambion, Victoria, Australia)/isopropanol precipitation or the RNeasy plant mini kit (Qiagen, Victoria, Australia) according to the manufacturer’s instructions. RNA concentration was measured using NanoDrop Microvolume spectrophotometer (ND-1000, ThermoFisher, New South Wales, Australia) and purity and integrity of RNA ensured based on both the A260 nm/A280 nm ratio (close to 2) and formaldehyde-agarose gel electrophoresis. TRIzol extracted total RNA was DNase treated with 5 units of RQ1 RNase-free DNase (Promega, Alexandria, New South Wales, Australia) for 30 min at 37 °C followed by phenol/chloroform extraction and ethanol precipitation. RNA was reverse-transcribed with SuperScript III Reverse Transcriptase (Invitrogen, Victoria, Australia) using oligo dT priming following the manufacturer’s instructions.

### 2.3. Expression Analysis by Semi-Quantitative RT-PCR and RT-qPCR

Semi-quantitative RT-PCR and RT-qPCR were performed using *Taq* F1 DNA Polymerase (Fisherbiotech, Wembley, West Australia, Australia) and EXPRESS SYBR GreenER (Invitrogen, Victoria, Australia), respectively, following the manufacturer’s instructions. RT-PCR was performed in 30 cycles using primers shown in Table S5. Amplicon products from RT-PCR were visualized on a 2% agarose/TBE gel. RT-qPCR was performed using Rotor Gene 6000 (Qiagen, Victoria, Australia). Relative transcript abundance was measured against the reference gene *FDH* using the comparative quantification method of the Rotor Gene Q Series software. All measurements were performed in technical triplicates. Student’s *t*-test was performed to determine significant (*p*-value < 0.05) differences in gene expression levels between control (Col-0 or L*er*) and mutants or between mock and *Fusarium oxysporum*-infected samples. All primer sequences are supplied in Table S5.

### 2.4. Sequence-Specific RT-PCR

One to two micrograms of total RNA was subjected DNase treatment using one unit of RQ1 RNase-free DNase followed by cDNA synthesis using both 0.2 µM forward and reverse primers in a 50 µL reaction containing (1X PCR buffer, 0.2 mM dNTP’s, 1.5 mM MgCl_2_, 0.2 µM primer(s), 12 U RNase out (Invitrogen), 60 U SuperScript III (Invitrogen) and 1.5 unit Platinum *Taq* DNA Polymerase). Following cDNA synthesis, the reaction was then subjected to PCR for 35 cycles. PCR products were visualized on a 3% NuSieve (Lonza, Victoria, Australia)/1X TBE gel.

### 2.5. Nuclear RNA-Immunoprecipitation 

Three grams of plant tissues (Aerial tissues of *Arabidopsis* seedlings) were collected from each genotype and subjected to cross-linking by formaldehyde. Cross-linked tissues were ground into fine powder in liquid nitrogen using a mortar and pestle, resuspended in Honda buffer (0.44 M Sucrose, 1.25% Ficoll, 2.5% Dextran T40, 20 mM Hepes KOH pH 7.4, 10 mM MgCl_2_, 0.5% Triton X-100, 5 mM DTT, 1 mM PMSF (Sigma-Aldrich, Castle Hill, New South Wales, Australia), 1% Plant protease inhibitors (Sigma-Aldrich), 8 U/mL RNase out (Invitrogen)) and filtered through two layers of Miracloth (Calbiochem-Novabiochem, New South Wales, Australia) to remove cellular debris and nuclei isolated through centrifugation. Isolated nuclei were lysed Nuclei lysis buffer (50 mM Tris-HCl pH 8.0, 10 mM EDTA, 1% SDS, 1 mM PMSF (Sigma-Aldrich), 1% Plant protease inhibitors (Sigma-Aldrich), 160 U/mL RNase out (Invitrogen)), followed by sonication on ice four times for 10 s at 25% amplitude with 1 min pauses. Nuclear lysate was isolated through centrifugation and diluted 10-fold in IP dilution buffer (1.1% Triton X-100, 1.2 mM EDTA, 16.7 mM Tris-HCl pH 8.0, 167 mM NaCl, 350 U/mL RNase out (Invitrogen)). The diluted lysate was pre-cleared for one hour with 25 µL of Salmon Sperm DNA/Protein A agarose beads (Millipore, Sydney, New South Wales, Australia) and immunoprecipitation was performed by incubating the pre-cleared lysate with 5 µg monoclonal anti-FLAG M2 antibody (F1804, Sigma-Aldrich) and 25 µL of Salmon sperm DNA/Protein A agarose beads (Millipore) for three hours at 4 °C.

Following immunoprecipitation, agarose-antibody-FLAG:AGO4/RNA complexes were washed three times in Wash buffer (150 mM NaCl, 20 mM Tris-HCl pH 8.0, 2 mM EDTA, 1% Triton X-100, 0.1% SDS, 1 mM PMSF (Sigma-Aldrich), 40 U/mL RNase out (Invitrogen), 5 U/mL RQ1 DNase (Promega, New South Wales, Australia)). FLAG:AGO4/RNA complexes were eluted with RNA-IP elution buffer (100 mM Tris-HCl pH 8.0, 10 mM EDTA, 1% SDS, 40 U RNase out (Invitrogen)) at 65 °C for one hour in the presence of 20 µg Proteinase K (Sigma-Aldrich).

**Figure 1:**
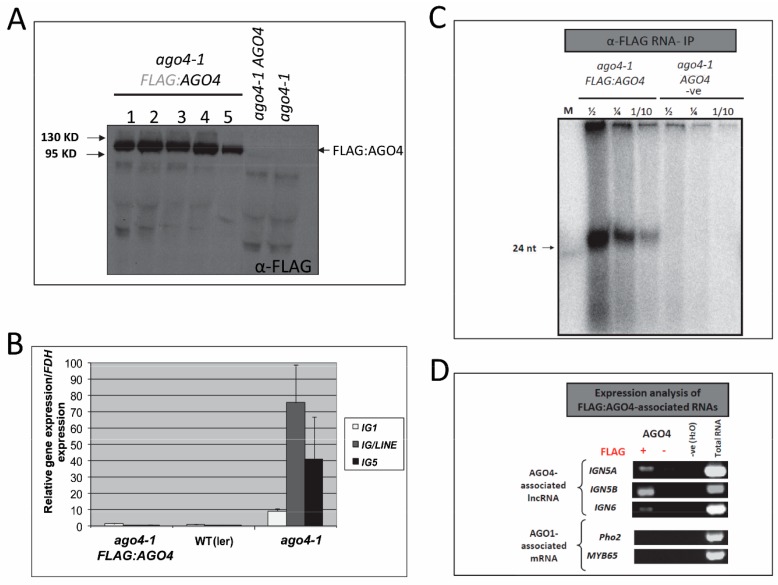
Analysis of transgenic FLAG:AGO4 expression and function, and specificity of FLAG:AGO4 RNA-immunoprecipitation (IP). (**A**) Western blot analysis of FLAG:AGO4 protein expression in transgenic *ago4-1 FLAG:AGO4* lines. *ago4-1 AGO4* and *ago4-1* plants were used as negative controls; (**B**) Real-time RT-qPCR analysis of the relative expression level of AGO4-regulated genes *IG1*, *IG/LINE* and *IG5* in wild type (L*er*), *ago4-1* mutant and transgenic *ago4-1 FLAG:AGO4* plants. Expression level with standard error (SE) shown is the average of 3 biological replicates, normalized to *FDH* (*Formate dehydrogenase*) and relative to L*er*; (**C**) Analyses of RNA quality derived from nuclear RNA-IP. RNA isolated by RNA-IP was labeled with radioactive ^32^P-ATP and separated by PAGE. ½, ¼ and 1/10 represent the amount of labeled sample loaded; (**D**) Expression analysis of specific AGO4-associated RNA (*IGN5A*, *IGN5B* and *IGN6*) and AGO1-associated RNA (*Pho2* and *MYB65*) in samples pulled down using nuclear RNA-IP.

Pulled-down RNA was purified by extraction with an equal volume of acidic phenol/chloroform (Ambion, Applied Biosystems, Victoria, Australia), followed by overnight ethanol precipitation in the presence of acidic sodium acetate and 20 µg Glycogen (Fermentas, Thermo fisher scientific, Victoria, Australia) at −80 °C, and resuspended in DEPC-treated H_2_O. RNA concentration was determined at 260 nm using NanoDrop Microvolume spectrophotometer (ND-1000, ThermoFisher). The quality of pulled-down RNA, as indicated by strong enrichment of AGO4-specific small RNA, was verified using 5’-end labeling with ^32^P-ATP and T4 polynucleotide kinase followed by sodium dodecyl sulfate (SDS)-polyacrylamide gel electrophoresis as shown in Figure 1C.

### 2.6. Chromatin-Immunoprecipitation Assay 

Chromatin-Immunoprecipitation (ChIP) was performed on 3-week old seedlings (1.5 g of aerial tissues) similar to RNA-IP except that no RNase inhibitor (RNaseOut) was used in all steps. Nuclear lysates were prepared as described above. Following nuclei lysis, nuclear lysates were saved in 25 µL aliquots at −80 °C. For immunoprecipitation, each lysate was diluted by 10-fold using IP dilution buffer and were pre-cleared for one hour with 50 µL of Salmon Sperm DNA/Protein A agarose beads (Millipore). Immunoprecipitation was performed by incubating the pre-cleared lysate with 2 µg anti-H3K27me3 (Millipore), 1.5 µg anti-H3K4me3 (Millipore) or 1.3 µg combination of anti-RNA Pol II (0.1 µg of 8WG16, 0.6 µg of phospho S5 and 0.6 µg of 4H8, Abcam) antibody and 50 µL of Salmon sperm DNA/Protein A agarose beads (Millipore) overnight at 4 °C.

Following immunoprecipitation, the immunoprecipitated complexes were washed five times in Wash buffer (150 mM NaCl, 20 mM Tris-HCl pH 8.0, 2 mM EDTA, 1% Triton X-100, 0.1% SDS, 1 mM PMSF (Sigma-Aldrich)) and twice with TE buffer. Immunoprecipitated complexes were reverse cross-linked in elution buffer (1% SDS, 0.1 M NaHCO3) at room temperature for 15 min under constant rotation, followed by incubation at 65 °C for four hours in the presence of 200 mM NaCl, and at 45 °C for one hour in the presence of 10 mM EDTA and 40 mM Tris-HCL pH 6.5 and 20 µg Proteinase K (Sigma-Aldrich). Pulled-down DNA was purified using the Qiagen PCR purification kit (Qiagen).

### 2.7. 5’ End Labeling and SDS-PAGE

RNA was labeled with ^32^P-ATP by incubation at 37 °C for 1 hour in a 20 µL reaction containing: 15–20 ng RNA, 1X T4 polynucleotide kinase (PNK) buffer, 5 µL of γ-ATP (250 uCi, Perkin Elmer, Victoria, Australia) and 10 U T4 PNK (Roche, NSW, Australia). Sample was purified through a Microspin G-25 column (GE Healthcare, NSW, Australia). Half (10 µL), one quarter (5 µL) and one tenth (2 µL) of the labeled and purified reaction was loaded and separated on a 20% SDS-PAGE. Radioactive signals were detected by phosphoimaging.

### 2.8. Template-Switch cDNA Library Preparation and Expression Analysis of AGO4- and AGO1-Associated RNA by PCR

150 ng of immunoprecipitated RNA (FLAG:AGO4 IP or –ve IP) or nuclei extracted RNA was converted to a cDNA library using the template-switch cDNA library preparation method according to Zhao et al. [24]. 1 µL from each cDNA library was used as template in a 30-cycle PCR reaction to detect AGO4-associated and unrelated AGO1-associated RNA. *IGN5A*, *IGN5B* and *IGN6* primers were derived from Wierzbicki et al. [16]. *Pho2* and *MYB65* primers (Table S5) were used to detect AGO1-associated RNA. Because of the low-level non-specific RNA in the negative control nuclear RNA-IP, the negative control IP library was amplified for an additional 15 cycles to generate adequate cDNAs for deep sequencing. As this study focused on longer noncoding RNA species, the higher molecular weight fraction of the cDNA library (200–500 bp) was gel purified (Figure S3) using Ultraclean DNA purification kit (MO BIO laboratories, Geneworks, South Australia, Australia) and used for Illumina deep sequencing, which should exclude the small RNA including the 24-nt siRNA sequences.

### 2.9. Deep Sequencing

All three cDNA libraries (FLAG:AGO4-RIP, negative RIP and nuclei RNA), prepared using the Template-switch cDNA preparation method (above), were subjected to single end 100 bp deep sequencing using the Illumina genome analyzer GAIIx (ACRF Biomolecular Resource Facility at John Curtin’s institute of medical research, Australian Capital Territory, Australia).

### 2.10. Bioinformatics

RNA-IP or RNA-seq reads were trimmed for adaptor sequences and low quality bases on ends and reads less than 30 nt were removed to prevent mapping 24 nt small RNA using the CLC genomics workbench (version 4.9) pipeline. Trimmed and filtered reads were mapped using the CLC genomics workbench RNA-seq analysis application allowing a minimum of 80% similarity in at least 70% of the read in order to account for single nucleotide polymorphism (SNP) differences between the experimental ecotype (Landsberg *erecta* or L*er*) against the sequenced *Arabidopsis* reference genome (Col-0, The *Arabidopsis* Information Resource version 10 or TAIR10); mapping up to 20 locations was allowed to avoid removing reads that map to repeat sequences. Reads density across chromosomes were generated using the MACS (Model-based Analysis of ChIP-Seq) peaks calling software [25] and visualized on Galaxy [26]. Because template-switch library construction and sequencing generate sequence from the opposite strand of the original RNA bound to FLAG:AGO4, all reads were considered as reverse complemented in subsequent analyses. Annotated sequences of protein-coding gene, transposons, pseudogenes, ncRNAs, miRNAs, snoRNAs, tRNAs were retrieved from the Commonwealth Scientific and Industrial Research Organisation (CSIRO) Division of Plant Industry Bioinformatics Group Genome Browser. Repeat sequences were retrieved from The Institute of Genome Research (TIGR) *Arabidopsis* Repeats database version 2.0.0. Analysis of read counts between annotations, gene features and 2 kb flanking regions were performed using Map2locifeatures application (Dr. Stephen Stuart, CSIRO Plant industry, Canberra, Australia) in which parameters were set to prioritize functional regions so that each read is accounted only once. Heat maps were generated using R (Version 2.14.2) using the ggplot2 package. Cufflinks was used for transcript assembly [27]. Identification of overlapping annotations between datasets was performed using the findsame script with Perl. Characterization of annotations was performed by gene ontology (GO) annotations and statistical analysis of gene group over-representation was performed using the Gene Ontology Analysis Toolkit for Agricultural Community (agriGO) [28].

### 2.11. Western Blot Analysis

Tissues were ground to fine powder under liquid nitrogen and resuspended in protein extraction buffer (50 mM NaPO_4_ pH 7.0, 10 mM EDTA, 0.1% Triton X-100, 0.1% Sarkosyl (N-laurylsarcosine) and 10 mM β-mercaptoethanol). Protein concentration was measured using the Bradford assay. 10 µg of proteins were separated on an 8% SDS polyacrylamide gel, transferred to Immobilon P membrane (Millipore) and detected by chemiluminescence using 1/1000 monoclonal anti-FLAG M2 antibody (F1804, Sigma-Aldrich) and 1/5000 anti-mouse Ig HRP conjugate (Chemicon, Victoria, Australia).

### 2.12. Bisulphite Sequencing

Genomic DNA extracted from Col-0 and *nrpe1* plants were subjected to bisulphite conversion using the method described in Wang et al. [29]. Nested PCR using specific primers (Table S5) was used to amplify the genomic fragment corresponding to *Cuff.5107* and *Cuff.705*, PCR products were gel purified using Ultraclean DNA purification kit and transformed into pGEM-T-Easy vectors (Promega). 15 to 16 clones for each PCR product were sequenced and analyzed using CyMATE (Cytosine Methylation Analysis Tool for Everyone) [30].

### 2.13. 5’ Rapid Amplification of cDNA Ends

5’ Rapid amplification of cDNA ends (RACE) was performed using the 5’ RACE System (version 2, Invitrogen) using random priming cDNA synthesis of three micrograms of total RNA extracted from Col-0 or *ago4-2* 3-week old seedlings, following the manufacturer’s instructions. Briefly, total RNA was reverse-transcribed using random primers followed by RNAse treatment and cDNA was purified using S.N.A.P. Column Purification. TdT Tailing of cDNA was used to add primer targets at the 5’ end of cDNA. Nested gene-specific primers (Table S5) were designed at the 3’ end of genes for PCR amplification of short truncated transcripts in combination with 5’ RACE primers provided by Invitrogen. For long transcripts (>1.5 kb), additional nested gene-specific primers were designed at the center of transcript.

### 2.14. Infection of Arabidopsis Plants with Fusarium Oxysporum

Growth of *F. oxysporum* f. sp. *conglutinans* was performed as previously described [31]. Three week-old plants were infected by dipping the roots in an 1 × 10^6^ spores/mL inoculum and replacing the plants on MS agar without sucrose. Plants were then grown under 16 h light–8 h dark photoperiod at 22 °C.

### 2.15. Data Access

Complete RNA-IP and RNA seq data sets generated from this publication have been deposited in NCBI’s Gene Expression Omnibus and are accessible through accession number GSE48617.

## 3. Results

### 3.1. Transgenic FLAG:AGO4 Functions Like Endogenous AGO4

In order to identify the population of RNAs associated with AGO4, a FLAG:AGO4 transgene, driven by the cauliflower mosaic virus 35S promoter, was transformed into *ago4-1* (*A. thaliana* ecotype Landsberg *erecta or* L*er*) mutant plants, resulting in transgenic lines that gave consistent expression of the FLAG:AGO4 protein (Figure 1A). To examine if the FLAG:AGO4 protein expressed from the heterologous 35S promoter followed a similar pattern of accumulation to the endogenous AGO4 protein, we first compared the expression pattern of the 35S promoter with that of the AGO4 promoter using the β-glucuronidase (GUS) gene as a reporter. GUS expression from the 35S promoter followed a similar pattern to the AGO4 promoter although with a higher expression level (Figure S1 and [13]). Furthermore, western blot analysis showed that FLAG:AGO4 protein accumulated in a tissue-specific manner despite being driven by the 35S promoter, with the highest level in flowers (Figure S2A) where 24-nt siRNA levels are relatively abundant [32,33]. This is in contrast to the accumulation of FLAG:AGO4 mRNA or the GUS protein, which showed relatively uniform levels across the different tissues (Figure S2B–E). This is consistent with the previous report suggesting that AGO4 protein accumulation depends on 24-nt siRNAs [13], and implies that the pattern of FLAG:AGO4 accumulation mimics that of the endogenous AGO4 protein, despite being expressed from the heterologous 35S promoter.

To establish whether the FLAG:AGO4 fusion protein maintains the function of endogenous AGO4, RT-qPCR was performed on three AGO4-regulated intergenic transcripts which were silenced in WT *Arabidopsis* but de-repressed in *ago4-1* mutant plants. As shown in Figure 1B, all the three known AGO4-targeted intergenic (IG) transcripts, *IG1*, *IG*/*LINE* and *IG5*, were re-silenced in the *ago4-1 FLAG:AGO4* plants, with their expression returned to the WT level. These results indicate that the FLAG:AGO4 fusion protein accumulates and functions like endogenous AGO4, and is capable of directing functional RdDM. 

### 3.2. Anti-FLAG:AGO4 RNA Immunoprecipitation Gives Highly Enriched AGO4-Specific RNA Species

Nuclear-specific function of AGO4 [34,35] enabled us to perform nuclear RNA-immunoprecipitation (RNA-IP), an improved RNA-IP method that minimizes background derived from cytoplasmic RNA [16,36]. The quality of the immunoprecipitated FLAG:AGO4-associated RNA was visualized by 5’ end labeling with radioactive γ-ATP and PAGE. As shown in Figure 1C, 24-nt AGO4-binding siRNAs were readily detectable in the FLAG:AGO4 immunoprecipitated samples, but were undetectable in control samples. Also, no signals of 21- or 22-nt sRNAs, associated with posttranscriptional gene silencing, were detectable in the FLAG:AGO4 IP. In addition to the 24-nt siRNA, high-molecular-weight RNA signals (>100 nt) were detected in the RNA-IP near the loading wells (Figure 1C); such larger RNA species could represent the lncRNAs that act as a scaffold to recruit RdDM machinery including the AGO4-siRNA complex to the target DNA [3,17]. Consistent with this, a semi-quantitative RT-PCR analysis showed clear enrichment of RdDM-associated lncRNAs over mRNAs in the RNA-IP (Figure 1D).

### 3.3. Establishment of an AGO4-Associated Non-Coding RNA Landscape by RNA-IP Seq

RNA-IP seq cDNA libraries were prepared using the template-switch cDNA library preparation method derived from Zhao et al. [24]. To enrich for lncRNAs and minimize the cloning of 24-nt siRNAs, PCR product of 200–500 bp in size (Figure S3) was selected for sequencing using the Illumina platform. As shown in Table 1, the FLAG:AGO4-IP library gave approximately 19 million reads after adaptor trim and size filtering, of which 84% were mapped to the *Arabidopsis* chromosomes 1 to 5, with only 0.3% mapped to the chloroplast and mitochondrial genomes. The negative control IP library had approximately 11 million reads, but in contrast to the FLAG:AGO4-IP library, the majority of the reads (75%) were mapped to the chloroplast/mitochondrial genome. This confirmed the high purity of the RNA-IP. Our additional control library, the nuclear RNA library, generated from total RNA extracted from isolated nuclei of *ago4-1 FLAG:AGO4* plants and used as the input RNA control did not show such dramatic bias for either the nuclear or the organelle genome. This suggests that all RNA species were well represented in the input library and the enrichment for RNAs derived from specific regions of the nuclear genome in the FLAG:AGO4 RNA-IP was a direct consequence of the pull-down. A clear enrichment in coding sequence reads in the input nuclear RNA-seq data over that of the FLAG:AGO4 RNA-IP seq data (Figure S4) further validates the reliability of our data. 

**Figure 2:**
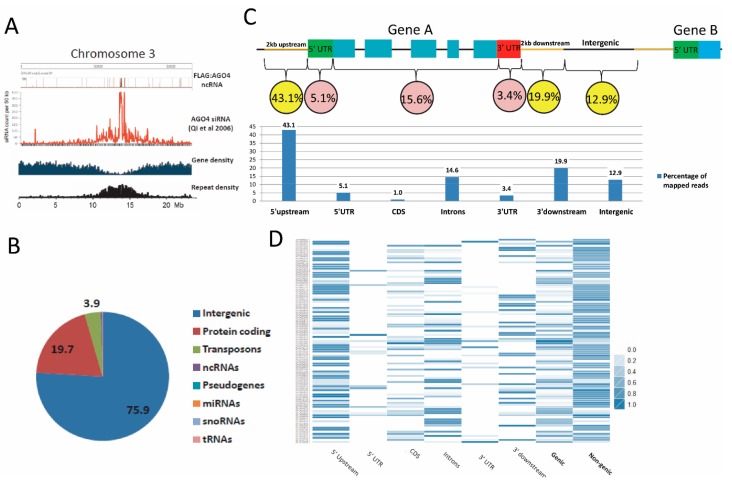
Bioinformatics analysis of FLAG:AGO4 RNA-IP seq data. (**A**) Chromosome wide density analysis of FLAG:AGO4 RNA-IP seq reads on Chromosome 3. Each peak represents a region on the chromosome with up to 5000 reads. A clear overlap in peaks between FLAG:AGO4 RNA-IP seq reads (>30 nt) and a published AGO4-associated 24 nt small interfering RNAs (siRNAs) data (Qi et al.) [15] is observed, particularly in regions that are high in repeats but low in genes; (**B**) Pie Chart illustration of read distribution across genomic features; (**C**) Analysis of read distribution across gene features (from 2 kb upstream to 2 kb downstream of annotated genes) shows preferential distribution of reads to non-coding regions; (**D**) Heatmap analysis of read distribution across gene features for 100 putative AGO4 target genes that carry at least 50 reads in the analyzed region (2 kb upstream to 2 kb downstream of an annotated gene). Values were transformed to Log_10_ scale and further normalized by ggplot2 package in R.

The high density regions of the FLAG:AGO4 RNA-IP reads showed a clear overlap with those of AGO4-associated 24-nt small RNAs derived from Qi et al. [15] (Figure 2A and Figure S5). These regions are low in genes but rich in repeats, which are known targets of RdDM. Indeed, 56.6% of the total mapped reads from the FLAG:AGO4 RNA-IP seq data match with repeat sequences (TIGR *Arabidopsis* Repeats database Version 2.0.0). By contrast, only 0.05% of the reads from the negative control RIP-seq library were repeat associated. Analysis of the mapped reads from FLAG:AGO4 RNA-IP seq against gene models showed that 75.9% originated from intergenic regions (including regions immediately upstream or downstream of genes), 19.7% from protein-coding genes and 3.9% from transposons (Figure 2B). This profile of read distribution is consistent with those derived from AGO4-associated small RNA libraries [33].

### 3.4. FLAG:AGO4 RNA-IP Seq Data Show Enrichment in RNA Reads from Noncoding Regions of Genes

To identify ncRNAs that might have a direct role in gene regulation, we scanned the FLAG:AGO4 RNA-IP seq reads that map to gene body and to the 2 kb upstream and downstream flanking regions. Parameters were set to prioritize functional regions so that each read is counted only once. Thus, if gene A is located within 2 kb upstream of gene B, reads that overlap with the body of gene A but also within the 2 kb upstream region of gene B are preferentially allocated to gene A. Reads in the intergenic region between gene A and gene B are separated from the mid-point and allocated to the 3’ downstream of gene A and the 5’ upstream of gene B, respectively.

Using this approach, we found that the reads, calculated as the percentage of the total number of mapped reads, were predominantly mapped to 2 kb 5’ upstream (43.1%) or 2 kb 3’ downstream (19.9%) flanking regions of genes and to the untranslated regions of the gene body (5’ UTR (5.1%), 3’ UTR (3.4%), introns (14.6%)), with only 1% of reads mapped to coding sequences (Figure 2C). In order to show that this distribution was not due to bias caused by abundant reads of only a few genes, a heatmap was generated to illustrate the distribution of reads across these genic and non-genic features at a single gene resolution. As shown in Figure 2D, read distribution was strongly biased to non-genic features. Thus, our FLAG:AGO4-RNA-IP seq is highly enriched for non-coding RNA reads, which suggests that RdDM regulates gene expression by targeting primarily non-coding regions. Furthermore, the majority of the RNA-IP seq reads is located in gene flanking regions, which is consistent with a previous study showing strong enrichment for 24-nt siRNAs and CHH methylation in gene ends in maize [37].

### 3.5. AGO4-Associated lncRNAs Overlap with AGO4 and POL V ChIP-Seq Data, TEs and AGO4-IP sRNAs

Cufflinks was employed to assemble the reads into ncRNA transcripts. This tool generated 18,953 genomic regions (Supplemental File 1) that produced AGO4-associated lncRNAs. Overlap of our AGO4-ncRNA data with previously published AGO4 [38] and POL V [39] ChIP DNA-seq data showed that 37% and 26% of ChIP peaks were identified in our data, respectively. To validate that this overlap was not due to chance, we generated a random dataset that mimics the size of both AGO4 and POL V ChIP peaks and found that only 15% of the random peaks overlap with our AGO4-associated lncRNAs library. Interestingly, only 9% and 12% of POL V and AGO4 ChIP peaks overlap with each other, respectively; this could be due to relatively high stringency of the Pol V and AGO4 ChIP datasets or alternatively, it could suggest that our data have identified a wider range of RdDM regulated regions that overlap with both ChIP data sets. To validate whether these lncRNAs could be involved in repressing transposable elements (TEs), we mapped TE sequences (obtained from TAIR) to the *Arabidopsis* genome to generate genomic TE coordinates. Then, we overlapped our data with these TE coordinates and found that 18% of these lncRNAs overlapped with TEs. However, when these AGO4-lncRNA contigs were extended by 500 bp in each direction, this overlap increased to 31% indicating that a substantial proportion of AGO4-associated lncRNAs are generated from genomic loci located in or in close proximity to TEs, consistent with the known role of RdDM in the repression of TEs. These extended contigs also overlap with 26%, 28%, 31% and 28% of *drm1/2* CHH, *drm1/2* CHG, *nrpdb2* CHH and *nrpdb2* CHG hypomethylated regions, respectively; and between 26 to 28% of *ago4*, *nrpd1* and *nrpe1* CHH or CHG hypomethylated regions [40]. To determine if these lncRNA transcripts are associated with AGO4 pull-down sRNAs, we downloaded deep sequencing data derived from AGO4-IP sRNAs in leaf, flower and seedling [33]. We mapped these AGO4-IP sRNAs (allowing up to two mismatches) to our AGO4-associated lncRNAs and found that 41.8%, 38.7% and 32.5% of our lncRNAs contain mapped AGO4-IP sRNAs derived from seedling, leaf and flowers, respectively. Collectively, 62.3% of our 18,953 AGO4-lncRNAs carry mapped AGO4-IP sRNAs generated by Wang et al. [33].

### 3.6. AGO4-Associated lncRNAs Show Partial Dependence on POL V for Transcription

**Figure 3:**
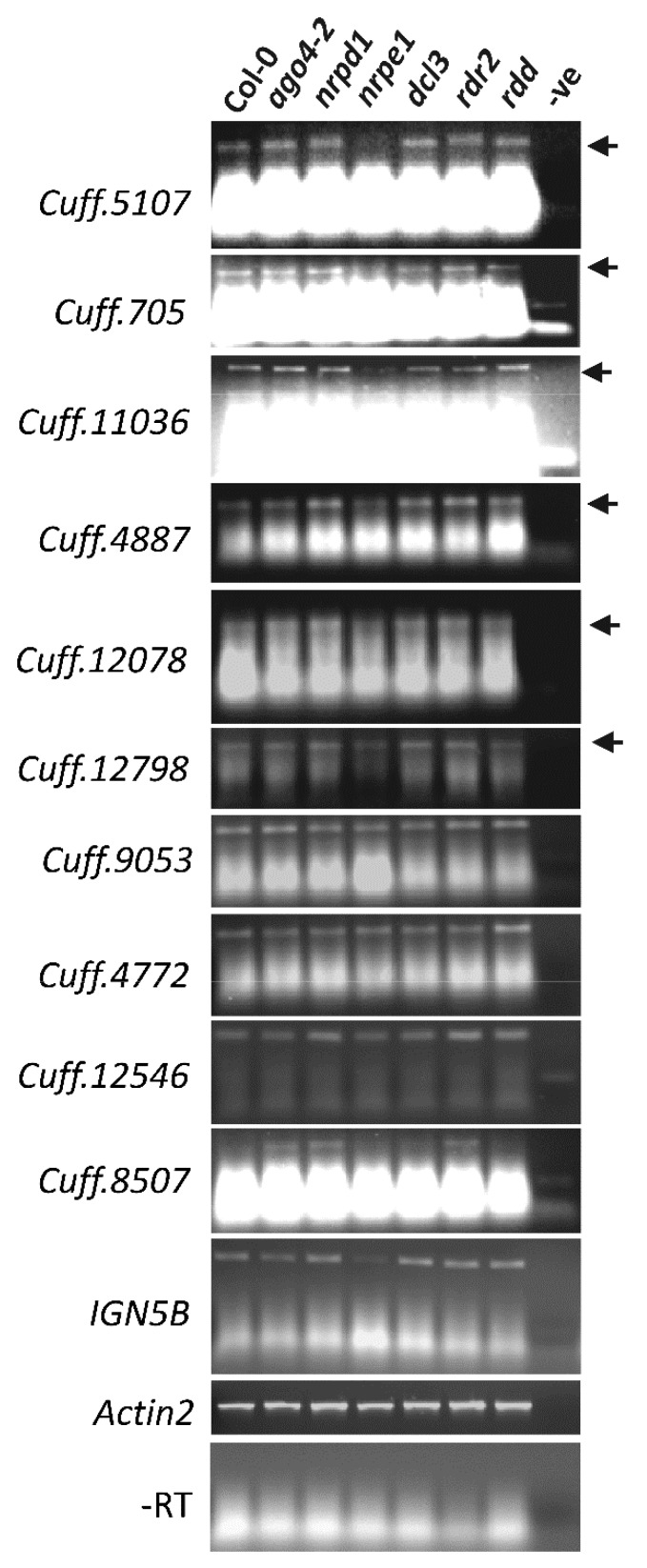
Expression analysis of FLAG:AGO4-associated long noncoding RNA (lncRNA). Semi-quantitative sequence-specific RT-PCR analysis of ncRNA expression in Col-0, RNA-directed DNA methylation (RdDM) mutants *ago4-2*, *nrpd1*, *nrpe1* and non-RdDM mutant *rdd* (triple demethylase mutant). *IGN5B* (a known POL V dependent lncRNAs), *Actin2* and –RT using *Cuff.5107* primers were included as controls.

In order to validate the lncRNAs, primers were designed to validate expression of lncRNAs in Col-0, RdDM mutants (*ago4*, *nrpd1*, *nrpe1*, *dcl3*, *rdr2*) and the non-RdDM mutant *rdd (ros1 dml2 dml3* triple demethylase mutant) using sequence-specific RT-PCR. lncRNAs were selected based on their level of enrichment in the Cufflinks dataset and their length (>150 bp) allowing better primer design. *IGN5B*, a known AGO4-associated and POL V-dependent lncRNA, was included as a control. As shown in Figure 3, nine of the 10 lncRNAs were expressed in WT Col-0, and three of these (*Cuff.5107, 705, 11036*) were clearly downregulated in the POL V mutant *nrpe1*, with another three (*4887, 12078, 12798*) also showing reduced amplification in *nrpe1* in comparison to the six control samples. This result suggests that Pol V is required for the transcription of these lncRNAs. However, the continued presence of low-level amplification for the six lncRNAs, and the lack of down-regulation for the remaining three lncRNAs, in the POL V mutant raise the possibility that some AGO4-associated lncRNAs are transcribed by other RNA polymerases such as POL II. This possibility is also suggested by the overlap between our AGO4-IP lncRNA sequences and the *nrpb2*-associated CHH and CHG-hypomethylated regions [40] as described above. Thus, full transcription of AGO4-associated lncRNAs may require not only Pol V but also other RNA polymerases.

### 3.7. nrpe1 Plants Show CHH Hypomethylation in Genomic Loci that Generate AGO4-Associated and POL V Dependent lncRNAs

**Figure 4:**
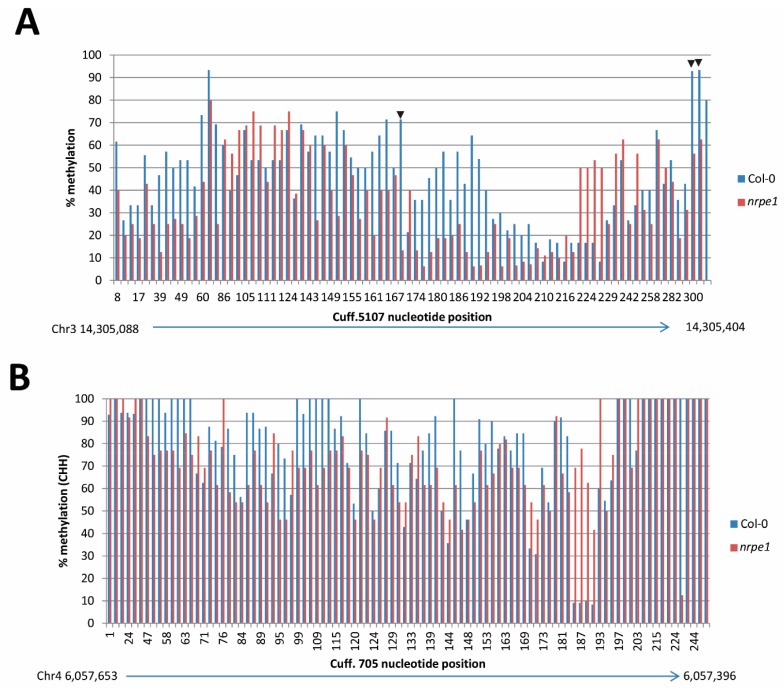
Analysis of bisulphite sequencing of *Cuff.5107* and *Cuff.705* loci. (**A**) CHG (arrowheads, three sites) and CHH (all other peaks, 81 sites) methylation level at potentially cytosine methylated positions across *Cuff.5107*; (**B**) CHH methylation (96 sites) level at potentially cytosine methylated positions across *Cuff.705*. Efficient bisulfite conversion was verified by amplifying a 157 bp chloroplast psaA protein gene sequence and digesting the PCR product with MseI restriction enzyme (Figure S6).

To validate the role of these POL V-dependent AGO4-lncRNAs in DNA methylation, bisulphite sequencing was performed to analyze the methylation status of genomic loci that generate *Cuff.5107* and *Cuff.705*, in Col-0 and *nrpe1* plants. Methylation analysis using CyMATE [30] identified seven (CG), three (CHG) and 81 (CHH); and two (CG), zero (CHG) and 91 (CHH) potential cytosine methylated sites in the genomic region covering *Cuff.5107* and *Cuff. 705*, respectively. This suggests that these regions are enriched in CHH methylation sites, consistent with the dominant CHH methylation characteristic of RdDM. While no change in CG methylation was observed between WT Col-0 and *nrpe1* (data not shown), CHG and CHH methylation was reduced in *nrpe1* plants. As shown in Figure 4, loss of POL V resulted in hypomethylation, predominantly at the CHH context (the hallmark of RdDM) in both genomic loci. Overall, 59 of the 81 CHH sites in *Cuff.5107* and 55 of the 96 CHH sites in *Cuff.705* showed reduced levels of methylation up to 87.5% in *nrpe1* plants. Some CHH sites showed a higher level of methylation in *nrpe1* in the two loci, but the number of such sites (17 and 23 CHH, respectively) is much smaller. This data validates the quality of our data in the identification of RdDM targeted loci.

### 3.8. The FLAG:AGO4 RNA-IP Seq Data Reveal Novel RdDM Target Genes

**Figure 5:**
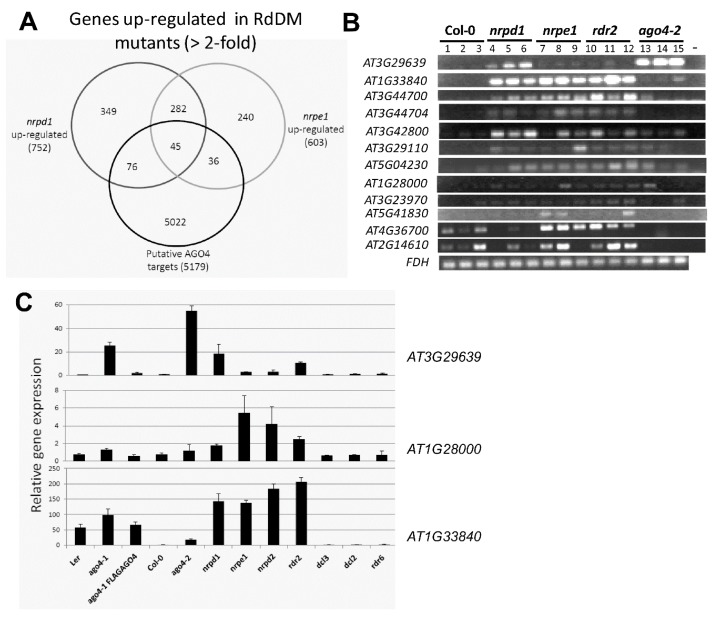
Identification and validation of putative RdDM-repressed genes. (**A**) Venn diagram illustration of overlapping common annotations between putative AGO4-targets identified based on the FLAG:AGO4 RNA-IP seq, and genes upregulated in RdDM mutants *nrpd1* (POL IV mutant) and *nrpe1* (POL V mutant) (>2-fold relative to Col-0) based on microarray data; (**B**) Semi-quantitative RT-PCR analysis of gene expression in wild type (Col-0) versus RdDM mutants (*nrpd1*, *nrpe1*, *rdr2* and *ago4-2*); (**C**) Real-time RT-qPCR analysis of three RdDM-repressed genes in plants of the L*er* ecotype (Wild type L*er*, *ago4-1*, transgenic *ago4-1 FLAG:AGO4*) and Col-0 ecotype (Wild type Col-0; RdDM mutants: *ago4-1*, *nrpd1*, *nrpe1*, *nrpd2*, *rdr2*, *dcl*3; and non-RdDM mutants: *dcl2* and *rdr6*). Expression level with SE shown is the average of 3 biological replicates, normalized to *FDH* and relative to Col-0.

A total of 5699 annotated genes were identified that had 50 or more reads corresponding to the gene body and 2 kb flanking regions; 5179 of these were protein-coding genes (Table S1). The presence of AGO4-associated ncRNAs suggests that these genes could be targeted by RdDM. To identify further RdDM targets from this list of genes, we searched an existing microarray gene expression dataset of two RdDM mutants *nrpd1* (POL IV mutant) and *nrpe1* (POL V mutant) (microarray data accession: GSE60508) [41] to identify genes that showed at least two-fold up-regulation over wild type Col-0. This analysis identified 45 genes (Figure 5A and Figure S7A, and Table S2) that had AGO4-associated ncRNAs and showed up-regulation in both of the RdDM mutants. A search of their expression pattern in the AtGeneExpress Visualization Tool database [42] showed that 35 of these genes are expressed across multiple tissue types in *Arabidopsis* (Table S2), suggesting that they are not pseudogenes.

To verify that these 45 genes are targeted and repressed by RdDM, we selected 12 genes and performed semi-quantitative RT-PCR and real time RT-PCR (RT-qPCR) to examine their expression in WT and RdDM mutant plants. Ten of the 12 genes showed no detectable expression in wild type (Col-0) plants but clear expression in at least two of the four RdDM mutants tested (*nrpd1*, *nrpe1*, *rdr2* and *ago4-2*) (Figure 5B), suggesting that these genes are usually repressed by RdDM. Of the ten genes, only *AT3G29639* is a known target of RdDM [9]. We analyzed the expression of three of these genes using RT-qPCR in additional RdDM mutants (*nrpd2* and *dcl3*), in plants of the L*er* ecotype (WT, *ago4-1*, transgenic *ago4-1 FLAG:AGO4*), and in non-RdDM mutants (*dcl2* and *rdr6*) as controls (Figure 5C). Gene expression was de-repressed in all the RdDM mutants in comparison to wild type plants (L*er* and Col-0) except in *dcl3*, which may be attributed to functional redundancy of DCL3 with other dicer-like (DCL) proteins. In contrast to the RdDM mutants, expression of the three genes did not change in non-RdDM mutants, confirming that these genes are specifically regulated by RdDM (Figure 5C). Furthermore, the RT-qPCR analysis showed that these three genes, which were de-repressed in *ago4-1*, were re-silenced by the expression of FLAG:AGO4 in the transgenic plants, suggesting that FLAG:AGO4 functions like endogenous AGO4 to repress these novel RdDM-regulated genes and further confirming that these genes are specifically targeted by RdDM.

### 3.9. RdDM Is Involved in the Activation of Stress-Responsive Genes

**Figure 6:**
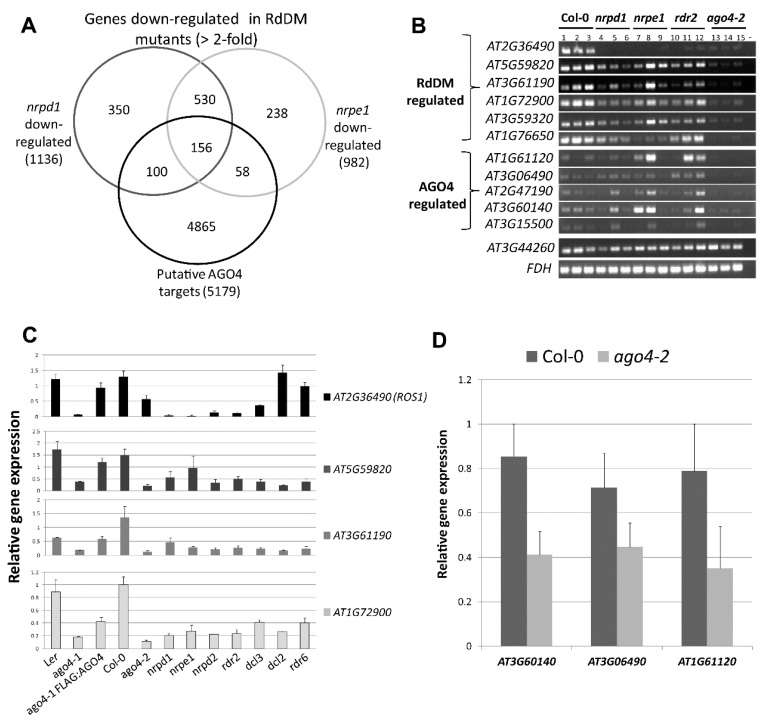
Identification and validation of putative RdDM-activated genes. (**A**) Venn diagram illustration of overlapping common annotations between putative AGO4-targets identified based on the FLAG:AGO4 RNA-IP seq, and genes downregulated in RdDM mutants *nrpd1* (POL IV mutant) and *nrpe1* (POL V mutant) (>2-fold relative to Col-0) based on the microarray data; (**B**) Semi-quantitative RT-PCR analysis of gene expression in wild type (Col-0) versus RdDM mutants (*nrpd1*, *nrpe1*, *rdr2* and *ago4-2*); (**C**) Real-time RT-qPCR analysis of four RdDM-activated genes in plants of the L*er* ecotype (Wild type L*er*, *ago4-1* and transgenic *ago4-1 FLAG:AGO4*) and Col-0 ecotype (Wild type Col-0; RdDM mutants: *ago4-1*, *nrpd1*, *nrpe1*, *nrpd2*, *rdr2*, *dcl*3; and non-RdDM mutants: *dcl2* and *rdr6*); (**D**) Validation of three AGO4-regulated genes by Real-time RT-qPCR in Col-0 versus *ago4-2*. Expression level with SE shown is the average of 3 biological replicates, normalized to *FDH* and relative to Col-0.

When comparing our FLAG:AGO4 RNA-IP seq data with the *nrpd1* and *nrpe1* microarray data, we identified 156 genes that had corresponding AGO4-associated ncRNA reads and were downregulated in *nrpd1* and *nrpe1* mutants by at least 2-fold (Figure 6A and Figure S7B, and Table S3). This number (156) is significantly larger than that of genes (45) upregulated in the RdDM mutants. This was surprising as RdDM is known to repress gene expression and RdDM-targeted genes are expected to be upregulated but not downregulated in the RdDM-deficient mutants. This result raises the possibility that RdDM may play a role in maintaining or activating the expression of protein-coding genes. Interestingly, a survey of expression patterns of these genes in the AtGenExpression Visualization Tool database [42] revealed an enrichment for preferential expression in the reproductive tissues. Of the 131 genes recorded in the database, 63 (48%) show strong preferential expression in floral tissues and seed compared to root and aerial vegetative tissues (Table S3). Such enrichment was not observed for the 45 genes upregulated in the *nrpd1* and *nrpe1* mutants (Table S2).

Functional classification of these 156 genes indicated a significant over-representation of genes associated with stress-response, with 28 of the 156 genes having a known or potential function in stress response (Figure S7B and Table S4). To examine if these genes are positively regulated by RdDM, we performed semi-quantitative or RT-qPCR on 12 of these stress-responsive genes in WT versus RdDM mutants. Eleven of the 12 stress-responsive genes showed reduced expression in the *ago4* mutant compared to WT plants, and six of them also showed downregulation in the RdDM mutants *nrpd1*, *nrpe1* and *rdr2* (Figure 6B,C), suggesting that they are under positive regulation by RdDM. One of these six genes was *ROS1* (*REPRESSION OF SILENCING*, or also known as *DML-1, DEMETER-LIKE PROTEIN 1*), a demethylase gene that is known to be positively regulated by RdDM [4,43]. In addition, RT-qPCR of four genes showed that the reduced gene expression level in *ago4-1* plants can be largely rescued by FLAG:AGO4 in *ago4-1 FLAG:AGO4* plants (Figure 6C), confirming that these genes are specifically targeted by AGO4-mediated RdDM.

As non-RdDM controls, we also tested the expression of the four genes in non-RdDM mutants including *dcl2* and *rdr6*. Expression of *ROS1* was not affected in *dcl2* and *rdr6*, suggesting that it is strictly regulated by RdDM. However, this was not the case for the other three genes (*AT5G59820*, *AT3G61190*, *AT1G72900*), which are also downregulated to various degrees in the non-RdDM mutants (Figure 6C). Currently we cannot explain how DCL2 and RDR6 might be involved in the regulation of these genes. However, there is new evidence that other non-canonical RdDM factors such as RDR6 and DCL4 can also function in concert with AGO4 to mediate RdDM [44]. An involvement of such AGO4-mediated non-canonical RdDM could explain why the stress response genes were in general more downregulated in the *ago4-2* mutant than in the *nrpd1*, *nrpe1* and *rdr2* mutants (Figure 6B–D).

### 3.10. RdDM/AGO4-Activated Stress-Response Genes Are Induced upon Fusarium Oxysporum Infection 

To understand the significance of these 28 stress response genes on plant immunity, an RNA-seq dataset generated by Zhu et al. [45], which investigated transcriptome changes in Col-0 plants at 1, 3 and 6 days post infection by the fungal wilt pathogen, *F. oxysporum*, was used to examine the changes in expression level of these stress-response genes upon *Fusarium* infection. As shown in Figure S8A, the majority (18) of the 28 genes were upregulated by at least 2-fold at one or more of the three time points, six genes did not show significant changes in gene expression, and five were downregulated particularly at 3 days post infection; some data were not available for all three time points. We used semi-quantitative RT-PCR to confirm the induction for 12 of the 15 stress-response genes by *Fusarium* infection (Figure S8B). The expression of five of these 12 genes was also analyzed using RT-qPCR, which again showed significant induction at the later infection stage (5 days post infection, Figure S8C). 

### 3.11. Repression or Activation of Gene Expression by RdDM May Depend on the Location of the Target Site in the Gene

**Figure 7:**
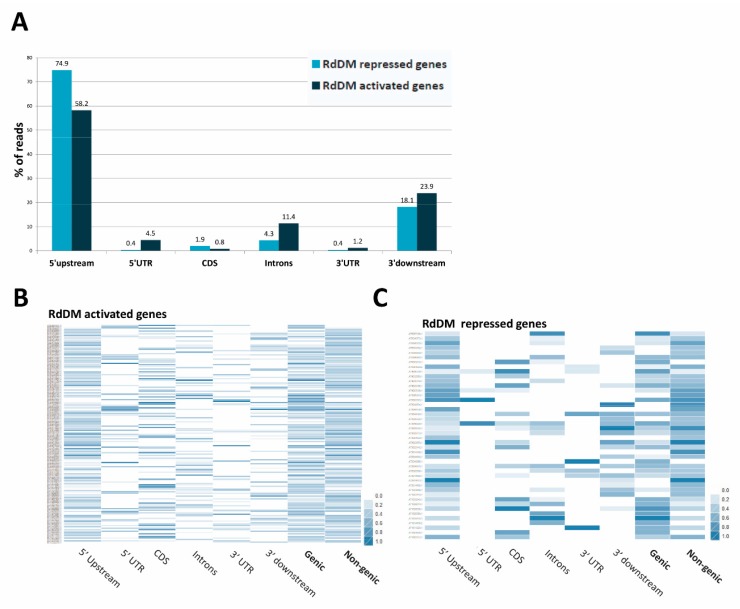
Read distribution in gene features of putative RdDM repressed and activated genes. (**A**) Comparison of overall read distribution in gene features between putative RdDM repressed and activated genes. Values are shown as the percentage of reads located in each gene feature over the total number of reads for that particular gene group; (**B**,**C**) Heatmap analysis of read distribution across gene features in 156 putative RdDM activated and 45 RdDM repressed genes. Values were transformed to Log_10_ scale and further normalized by ggplot2 package in R.

A comparison of ncRNA read distribution across gene features between the putative RdDM repressed (45 genes) and activated genes (156 genes) revealed a different pattern in ncRNA distribution. The repressed genes had more reads mapped to the upstream flanking sequence than the activated genes. In contrast, activated genes showed more ncRNA reads than the repressed genes along the gene body, particularly in the intronic region where the difference is almost three fold (Figure 7A). There was increased read distribution in the gene body for the activated genes as indicated by a more evenly distributed heatmap between the genic and non-genic region (Figure 7B). This is in contrast with the heatmap shown in Figure 2D where genes were not selected based on expression phenotype. Additionally, heatmap analysis of RdDM repressed genes continued to show a preference for the non-genic region (Figure 7C), although the relatively small number of genes makes the comparison difficult to interpret.

An enrichment of ncRNA reads in the upstream region in the repressed genes is consistent with promoter methylation being involved in transcriptional repression of gene expression. The enrichment of ncRNA reads in the gene body region of activated genes, on the other hand, could suggest that RdDM occurs in the gene body to maintain or activate gene expression. This scenario would be consistent with previous reports showing a positive correlation between gene body methylation and gene expression levels in *Arabidopsis* [46]. Shibuya et al., recently showed that in the flowering plant *Petunia hybrida*, targeting the intron of the *MADS3* gene for methylation by RdDM can result in up-regulation of gene expression [47].

### 3.12. AGO4-Associated lncRNAs Derived from Gene Body Are Associated with H3K4me3 Marks

**Figure 8:**
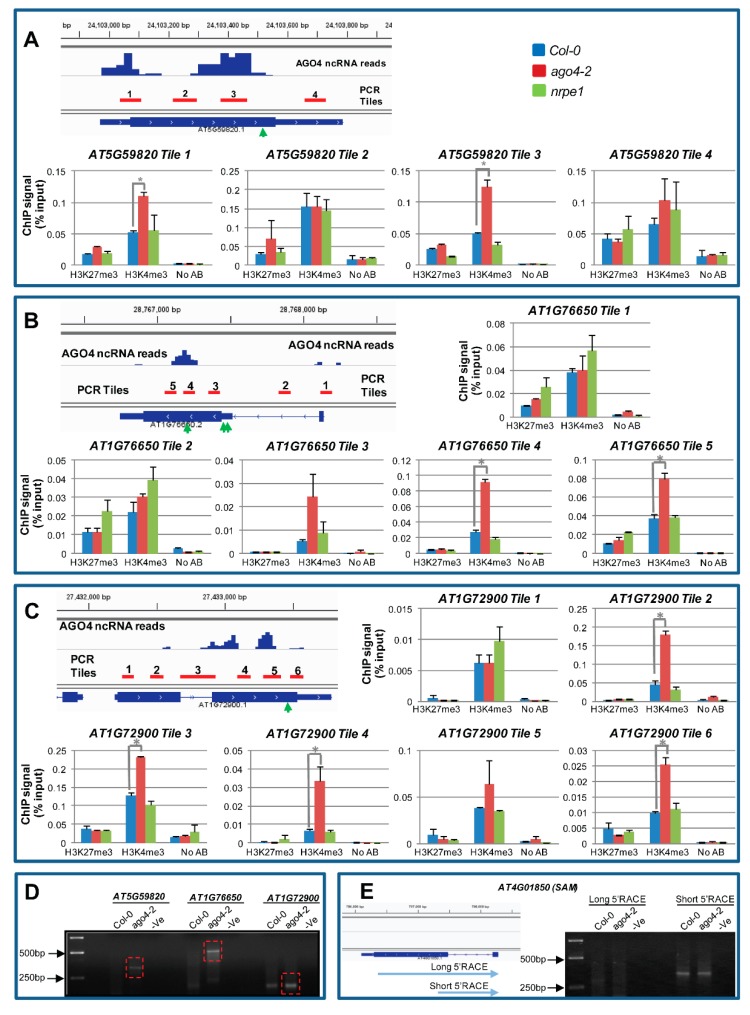
Chromatin immunoprecipitation (ChIP) assay and 5’ RACE detect increased H3K4me3 marks and short transcripts inside the gene body of RdDM-activated genes in the AGO4 mutant. Histone H3 Lys 27 (H3K27me3) and Lys 4 (H3K4me3) trimethylation at gene body tiling regions of (**A**) *AT5G59820*, (**B**) *AT1G76650* and (**C**) AT1G72900. ChIP signals are normalized to the input signals. Error bars are the standard error of the mean from two biological replicates. Green arrows show the start site of 5’ RACE products cloned from *ago4-2* tissues. Student’s *t*-test is used to detect significant differences at the *p* < 0.05 level (*); (**D**) 5’ RACE product, representing short RNA transcript initiated inside the gene body, is readily detectable in *ago4-2* (boxed in red), but undetectable or accumulates at lower levels in WT Col-0; (**E**) No such 5’ RACE product initiated inside the gene body (long 5’ RACE) is detectable in either *ago4-2* or Col-0 for the control gene *AT4G01850*. The short 5’ RACE product represent full-length transcript of the gene.

Next, we searched the TAIR genome browser for evidence of DNA methylation in the gene body of RdDM regulated active genes and found that of the six RdDM activated genes verified by RT-PCR in Figure 6C, only two (*AT2G36490* and *AT3G59320*) have methylation exclusive to the gene body, which is predominantly CG methylation. The remaining four genes (*AT5G59820*, *AT3G61190*, *AT1G72900* and *AT1G76650*) had no DNA methylation in the gene body or in gene flanking regions according to the TAIR genome browser. Bisulphite sequencing of genomic regions in the gene body of these four genes that produced lncRNAs again showed no evidence of methylation (data not shown). We then searched for evidence of changes in histone modification marks across the body of these genes. As shown in Figure 8A–C and Figure S9, the gene bodies of all four genes are associated with H3K4me3 marks, the hallmark of active gene transcription, consistent with their expression in WT Col-0 plants. Furthermore, these active histone marks increased in *ago4-2* mutants specifically in or near the regions overlapping with lncRNAs. This appears to contradict the fall in mRNA expression level of these genes in *ago4-2* as shown in Figure 6B,C, but transcription repression of these genes in *ago4-2* were further indicated by the reduced RNA Polymerase II (Pol II) occupation at their transcription start site as measured by the Pol II ChIP assay (Figure S10). Because H3K4me3 regulates gene expression predominantly at the 5’ end of genes [48] and the observed increase in H3K4me3 in *ago4-2* is far from the 5’end of genes; we speculate that these active histone marks may reflect increased expression of cryptic transcription units inside the gene body that interferes with the normal transcription of the hosting gene, and that AGO4 may play a role in repressing this cryptic transcription thereby maintaining or activating the expression of the gene in which the lncRNA occurs. Consistent with this possibility, a 5’ RACE experiment detected short RNA transcripts in *ago4-2* for all three RdDM-activated genes analyzed (*AT5G59820*, *AT1G76650*, *AT1G72900*) (Figure 8D). These transcripts were either undetectable (for *AT5G59820* and *AT1G76650*), or accumulated at a lower level (for *AT1G72900*) in the wild-type Col-0 plant. Such increased accumulation of short RNA transcripts did not occur in *ago4-2* for the control gene (*AT4G01850*) that does not have AGO4-associated lncRNAs in the gene body (Figure 8E). Sequencing of the 5’ RACE products showed that the short transcripts from two of the RdDM-activated genes started immediately downstream of the dominant AGO4 lncRNA peak regions (Figure 8A for *AT5G59820* and Figure 8C for *AT1G72900*; see the green arrow for starting sites). This suggests that the lncRNA-overlapping regions may represent cryptic promoters and that RdDM may play a role in repressing the cryptic promoters in wild-type plants preventing the production of the downstream short transcripts.

It is worth noting that in contrast to the AGO4 mutant, H3K4me3 was not affected in the POL V mutant (*nrpe1*, Figure 8 and Figure S9). The down-regulation of these genes (*AT5G59820*, *AT3G61190*, *AT1G72900* and *AT1G76650*) was also stronger in *ago4-2* compared to *nrpe1* (Figure 6B,C). This suggests that disrupting AGO4 function can cause more severe epigenetic changes than mutation of other canonical RdDM factors. Consistent with this, recent studies showed that while *ago4* mutant is susceptible to the bacterial pathogen *Pseudomonas syringae* [19], *nrpe1* is resistant [20], suggesting that AGO4 has targets outside of the canonical RdDM pathway.

## 4. Discussion

The principal aim of the present study was to identify genes that are targeted by RdDM, particularly those which may play a role in plant disease resistance. We performed transcriptome analysis by RNA-IP pull-down of AGO4-associated lncRNA followed by deep sequencing to identify gene loci regulated by RdDM in the *Arabidopsis* genome. We chose AGO4 as the bait for the RNA-IP pull-down instead of POL V because lncRNAs involved in RdDM may not be derived just from POL V transcription but could also come from Pol II transcription, as suggested by Zheng et al. [49]. We established a highly AGO4-specific lncRNA library as illustrated by low levels of protein-coding sequences and high abundance in non-coding sequence reads, most of which map to repeat-associated sequences and overlap with an AGO4-siRNA dataset from a separate study. In addition, the specificity of the FLAG:AGO4 RNA-IP seq data was evaluated by comparing the genome wide read distribution against the control negative RNA-IP seq and the input nuclear RNA library.

RdDM that occurs in both gene body and flanking regions could potentially regulate the expression of the corresponding genes. Therefore, to identify RdDM-targeted genes, we searched the RNA-IP seq data for ncRNAs that overlap not only with the gene body but also with 2 kb upstream and 2 kb downstream regions. We found that the majority of these ncRNA reads were derived from the flanking regions and the untranslated sequences of the gene body; only 1% was mapped to coding sequences. These suggest that the regulation of genes through RdDM occurs predominantly in non-coding regions particularly at promoters.

A total of 5179 genes were identified associated with the FLAG:AGO4 RNA-IP seq data, suggesting that a large number of genes are potentially regulated by AGO4 or RdDM. By overlapping the RNA-IP seq data with the microarray expression data of the POL IV (*nrpd1*) and POL V (*nrpe1*) RdDM mutants, 45 putative RdDM-target protein-coding genes were identified. This relatively small number could suggest that not all AGO4-regulated genes share the same dependency on POL IV and POL V, and some of the ncRNAs associated with AGO4 may be transcribed by other RNA polymerases. Consistent with this possibility, a recent report identified RdDM targets in which POL V-dependent lncRNAs can also be transcribed by RNA POL II [49]. This is also evident in our lncRNA validation in which not all lncRNAs are downregulated in *nrpe1* plants. Furthermore, Kurihara et al., identified RdDM repressed genes which show low dependency on RNA POL IV and V but high dependency on other RdDM factors RDR2 and DDC (DRM1 DRM2 CMT3) [9]. Thus, it is possible that genes that are regulated by AGO4-mediated RdDM but which are independent of RNA POL IV or V may have escaped our AGO4 lncRNA-microarray expression overlapping analysis.

The overlapping analysis between the FLAG:AGO4 RNA-IP seq data and the *nrpd1* and *nrpe1* microarray expression data also identified genes that are downregulated in the POL IV and POL V mutants. The number of genes in this category, 156, is larger than that of the genes upregulated in the mutants. This suggests that AGO4-mediated RdDM may potentially function as a positive regulator of gene expression and that genes can either be repressed or activated by RdDM. Interestingly, the 156 RdDM-activated genes are enriched for preferential expression in the floral tissues (Table S3), which coincides with the relatively high abundance of 24-nt siRNAs [32,33] and AGO4 protein (Figure S2A and [13]) in the flowers. This further suggests that these genes are positively regulated by RdDM.

A comparison of ncRNA read distribution between genes repressed by RdDM and those activated by RdDM show that the latter possess a higher proportion of reads that map to the gene body, suggesting that gene body methylation through RdDM may potentially maintain/activate gene expression. Gene body methylation has been observed in both plants and many other eukaryotic organisms including *Ciona intestinalis* (sea squirt), the silkworm [50] and human [51,52,53]. Recent genome-wide DNA methylation analysis has shown a positive correlation between high-level gene expression and gene body methylation in *Arabidopsis* [18]. However, analysis of four RdDM-activated genes detected no gene body DNA methylation in WT *Arabidopsis* or *ago4* mutant plants. Instead, these four genes showed the active histone mark, H3K4me3, in the gene body regions that overlap with the lncRNAs, and the level of this active histone mark is increased in the *ago4* plants. This raises the possibility that AGO4-mediated RdDM can function through histone modification independently of DNA methylation, and that the role of RdDM in the gene body is to repress cryptic transcription. It has recently been speculated that cryptic transcription inside gene body can inhibit the proper expression of the gene and a function of gene body methylation is to repress such cryptic transcription [54]. A recent study on TE sequences inside introns of genes in *Arabidopsis* showed that intronic TEs of actively expressed genes are still targeted by RdDM and the heterochromatic (or repressed) state of these gene body TEs is critical for proper transcription of associated genes [55]. These and our findings collectively support the idea that RdDM directed against the gene body functions to maintain active gene expression by repressing cryptic transcription. Increased levels of cryptic transcription around the lncRNA sites in *ago4-2* plants is supported by the detection of short RNA transcripts in *ago4-2* with alternative start sites around or downstream of the lncRNA regions in the RdDM-activated genes analyzed. The decrease in mRNA level and Pol II occupation of the genes in *ago4-2* plants suggests that this cryptic transcription inside the gene body can inhibit gene expression. Several recent studies have shown that some RdDM target loci that are devoid of DNA methylation footprint show positive changes in active histone marks in RdDM mutants. Lopez et al. [20] showed that the promoter of disease defense gene *PATHOGENESIS-RELATED GENE 1* (*PR-1*) has increased H3K4 trimethylation and/or H3K9 acetylation marks in RdDM mutants of *nrpd1* and *nrpe1*; but is devoid of DNA methylation. Wierzbicki et al. [39] showed that POL V target *AT1G26250* that lacks the CHH methylation mark is more actively acetylated at the H3K9 and H3K14 residues in *nrpe1* mutants. These and our results suggest that RdDM factors can modulate changes in gene expression through changes in histone marks independent of DNA methylation and therefore point to the existence of an alternative RNA-directed chromatin modification pathway in plants.

The putative RdDM-activated genes were enriched for stress-response function, including *ROS1*, a demethylase gene whose expression level is known to be positively regulated by RdDM. Surprisingly, three of four of the RdDM-activated genes analyzed using RT-qPCR showed down-regulation not only in the RdDM mutants but also in the PTGS mutants *dcl2* and *rdr6*. Recent studies have indicated that *Arabidopsis* has a second siRNA-dependent DNA methylation pathway that requires the PTGS factors such as RDR1/6 and 21 nt siRNAs, and targets a subset of non-conserved genomic loci [56,57]. Therefore, the RdDM-activated genes may be regulated by both the canonical and the PTGS-associated RdDM pathways.

Eighteen of the RdDM-activated stress response genes showed increased expression levels in plants infected with *F. oxysporum* compared with uninfected samples. This result implies that RdDM may be enhanced during fungal infection to maintain or activate the expression of important stress-response genes, conferring an increased level of disease resistance. This scenario would be consistent with the RdDM mutants *ago4* and *nrpe1* being highly susceptible to bacterial or fungal pathogens as reported recently [20]. It is also consistent with the enhanced *F. oxysporum* resistance shown by the FLAG:AGO4 and AGO4:FLAG transgenic *Arabidopsis* plants (Figure S11). Recent evidence suggests that upon biotic stress, the *Arabidopsis* genome undergoes extensive DNA methylation changes [58]. Based on our data and those derived from recent publications, we propose that upon biotic stress, RdDM, in concert with other methylation and demethylation pathways, functions to fine-tune changes at the chromatin level to restore proper gene expression, particularly in stress-response genes to confer resistance against various infections. However, if and how RdDM is activated by stresses and how RdDM controls gene expression through gene body DNA/chromatin modifications remains to be further investigated.

## 5. Conclusions

Using nuclear RNA-IP seq of the AGO4-associated RNA transcriptome, we have generated a high-quality ncRNA dataset that will serve as a useful resource for identification of novel RdDM-targeted genes. We used this dataset to identify a number of novel RdDM target genes that may play a role in plant stress response. Furthermore, we present compelling evidence supporting the notion that in addition to gene repression, RdDM can positively regulate expression of genes associated with plant immunity, which could account for the increased disease susceptibility observed in RdDM mutants. It is, however, undeniable that the complexity of RdDM is beyond what we have presented here and more effort is required to understand RNA-induced DNA methylation and its function(s) in plants.

## Figures and Tables

**Table 1:** Summary of read distribution across the *Arabidopsis* genome in FLAG:AGO4 RNA-IP seq, negative control RNA-IP seq and input control nuclear RNA seq libraries. Negative control RNA-IP seq library was amplified by an additional 15 PCR cycles. Chr C, chloroplast genome; Chr M, mitochondria genome.

	FLAG:AGO4 RNA-IP	Negative RNA-IP (+15 Cycles)	Nuclear RNA
# reads after adaptor trim	18,878,339	10,799,912	17,537,595
# reads map to Chr 1-5	15,905,349 (84%)	1,071,294 (13%)	6,307,354 (36%)
# reads map to Chr C & M	50,238 (0.3%)	8,087,305 (75%)	3,467,242 (20%)
% total mapped reads	84.3%	88%	56%

## Supplementary Materials

The following are available online at www.mdpi.com/2073-4425/8/8/198/s1. Figure S1. The 35S and AGO4 promoters show a similar pattern of expression in *Arabidopsis*. Figure S2. The accumulation of FLAG:AGO4 protein is tissue type-dependent. Figure S3. Illustration of template-switch generated cDNA library of FLAG:AGO4 and negative RNA-IP RNA on 3% Nusieve 3:1 agarose gel. Figure S4. Read distribution across loci features between FLAG:AGO4 RNA-IP and nuclear RNA seq libraries. Figure S5. Chromosome wide density analysis of FLAG:AGO4 RNA-IP seq reads on Chromosome 1, 2, 4 and 5. Figure S6. MseI digestion confirms efficient bisulfite conversion of cytosines in the Col-0 and *nrpe1* samples used for bisulfite sequencing methylation analysis in Figure 4. Figure S7. GO annotation analysis of RdDM repressed (A) and activated (B) genes according to biological function. Figure S8. Expression analyses of putative RdDM-regulated stress response genes upon infection by *Fusarium oxysporum*. Figure S9. H3K27me3 and H3K4me3 ChIP assay. Figure S10. Pol II ChIP assay shows reduced Pol II occupancy in the transcription start site (TSS) of RdDM-activated genes in the AGO4 (*ago4-2*) mutant. Figure S11. Transgenic *Arabidopsis* plants over-expressing AGO4 show enhanced resistance to *Fusarium oxysporum (F. ox)*. Supplemental file 1. Cufflinks assembled AGO4-ncRNA transcripts. Table S1. 5699 annotated genes with 50 or more reads in gene body or 2 kb flanking regions. Table S2. List of 45 RdDM repressed genes, annotations up-regulated in nrpd1 and nrpe1 by => 2-fold which carry AGO4 lncRNA in the gene or 2kb upstream or downstream gene flanking regions. Table S3. List of 156 RdDM activated genes, annotations down-regulated in nrpd1 and nrpe1 by => 2-fold which carry AGO4 lncRNA in the gene or 2kb upstream or downstream gene flanking regions. Table S4. Summary of response to stimulus genes down-regulated in nrpd1/nrpe1 and with AGO4-associated ncRNA. Table S5. Primers used in this study.
