# Supplementary material for: Analysis of Argonaute 4-Associated Long Non-Coding RNA in Arabidopsis thaliana Sheds Novel Insights into Gene Regulation through RNA-Directed DNA Methylation
Source: Genes (Basel). 2017 Aug 7;8(8):198. doi: 10.3390/genes8080198 (PMC5575662; doi:10.3390/genes8080198)
Supplement: Supplementary file 1 [file genes-08-00198-s001.zip › Supplementary materials/Supplemental Figures.pdf]

Supplementary Figures

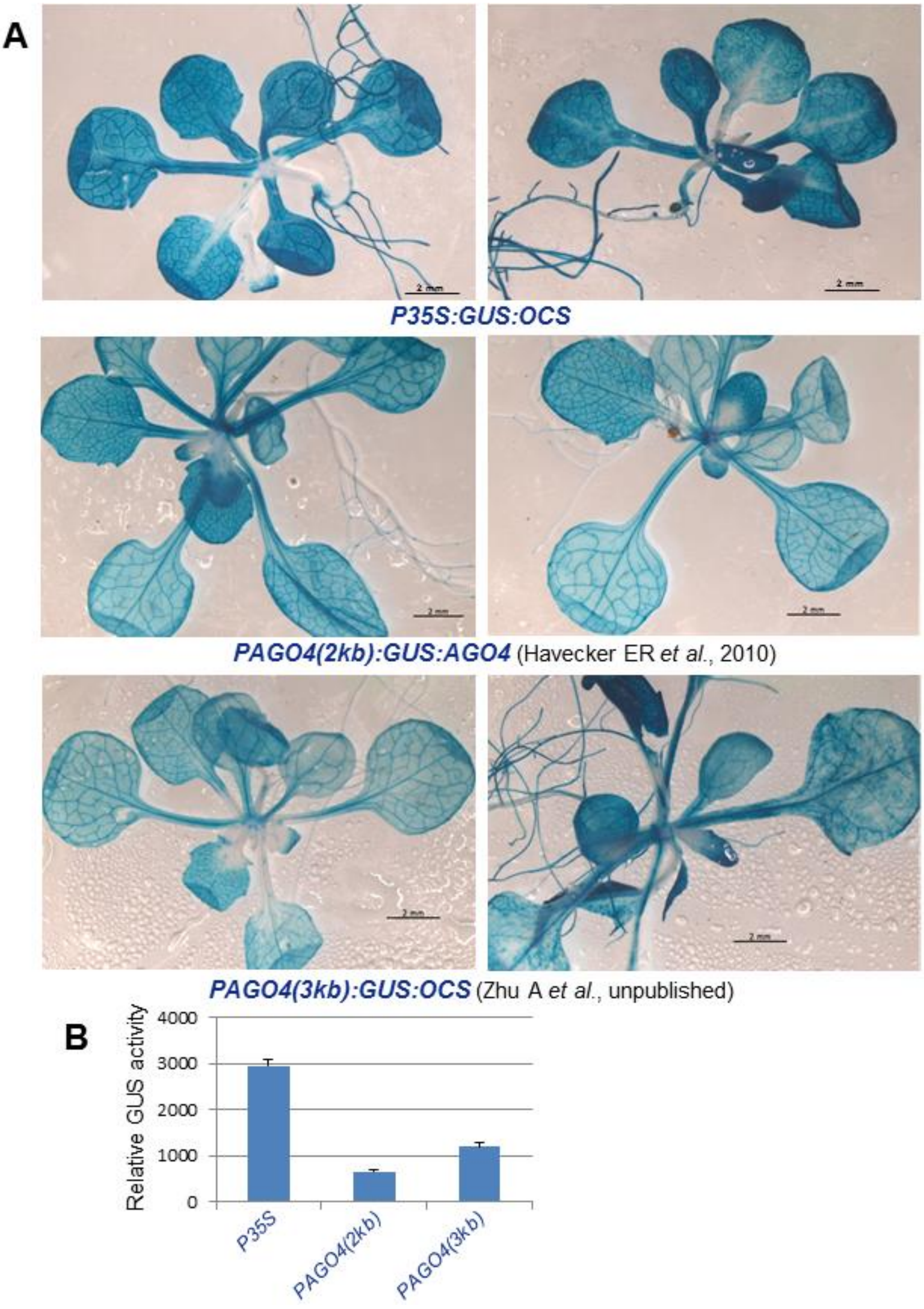

Figure S1. The 35S and AGO4 promoters show a similar pattern of expression in Arabidopsis. (A) Histochemical GUS staining showing a similar constitutive expression pattern between the *P35S:GUS* and *PAGO4:GUS* transgenes. (B) The 35S promoter shows a higher level of expression than the AGO4 promoter. OCS, *Agrobacterium tumefaciens* Ti plasmid octopine synthase gene terminator.

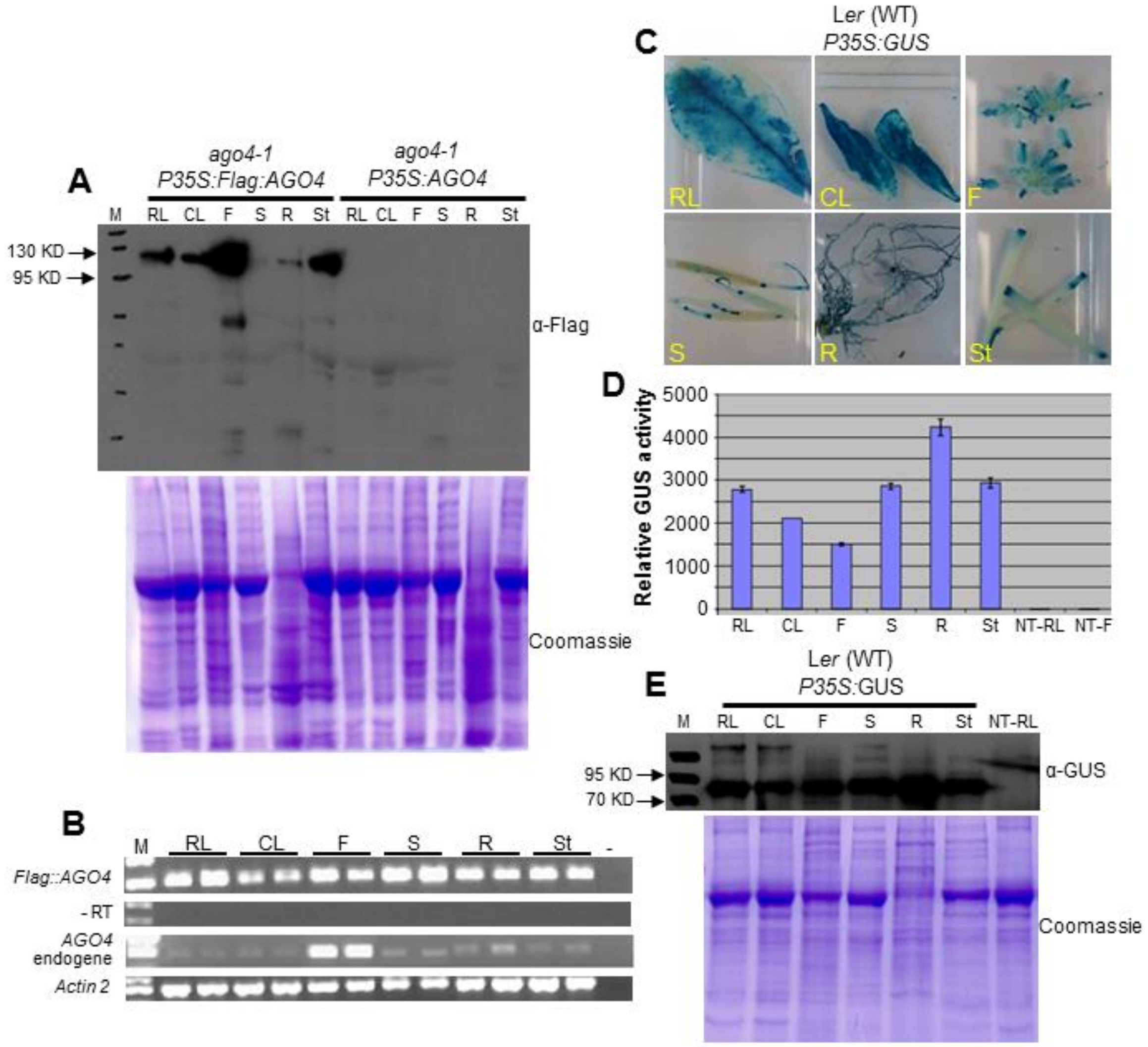

Figure S2: The accumulation of FLAG:AGO4 protein is tissue type-dependent. Left panels: FLAG:AGO4 protein accumulates at high abundance in immature flowers and stems, and low levels in leaves and roots (A), in contrast to FLAG:AGO4 mRNA, which accumulates relatively uniformly across different tissues (B). Right panels: The accumulation of GUS protein is not highly dependent on tissue type. The amount of GUS activity or GUS protein, as determined by histochemical X-Gluc (5-bromo-4-chloro-3-indolyl b-D-glucuronide) staining (C), fluorometric MUG (4-methylumbelliferyl-beta-D-glucuronide) assay (D), and Western blotting (E) show relatively uniform accumulation across different tissues. P35S, caulifower mosaic virus 35S promoter; RL, rosette leaves; CL, cauline leaves; F, flowers; S, sliques; R, roots; St, stems; NT, non-transgenic Arabidopsis.

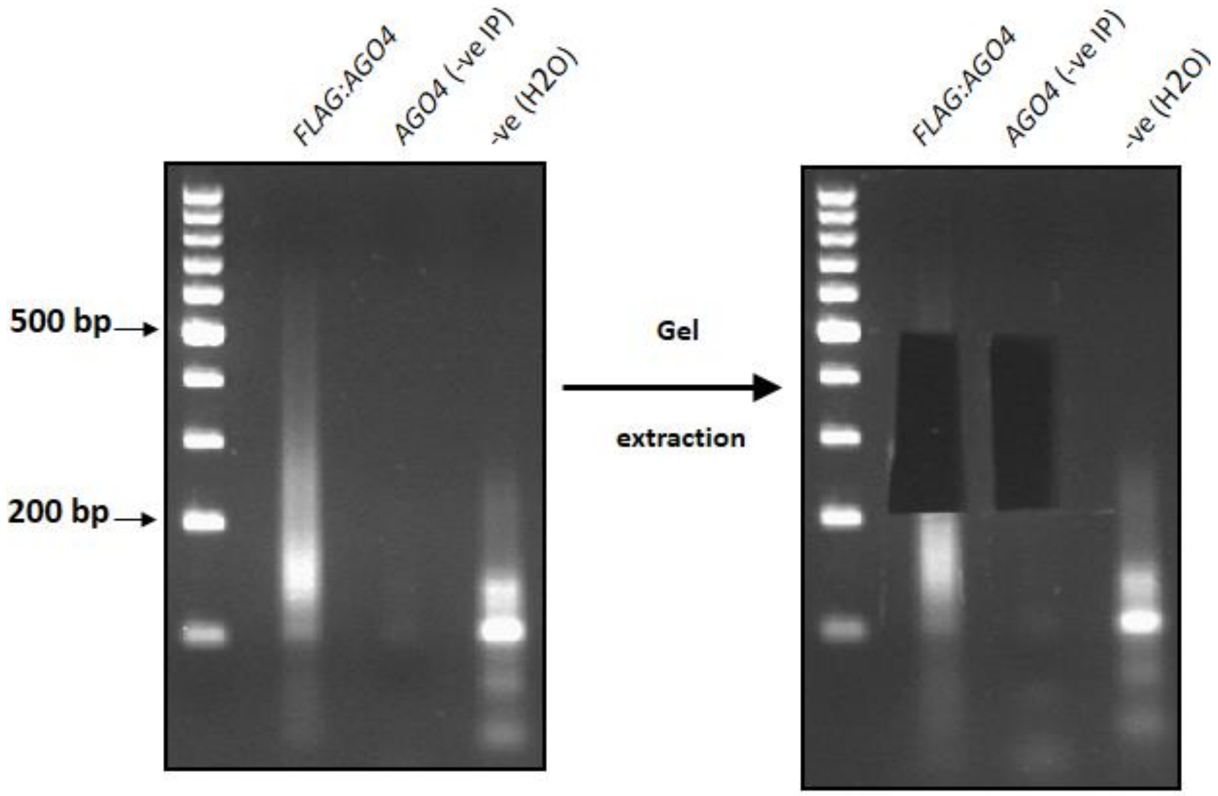

Figure S3: Illustration of template-switch generated cDNA library of FLAG:AGO4 and negative RNA-IP RNA on 3% Nusieve 3:1 agarose gel. cDNA library ranging from 200 to 500 bp from each sample were gel extracted and purified for Illumina sequencing.

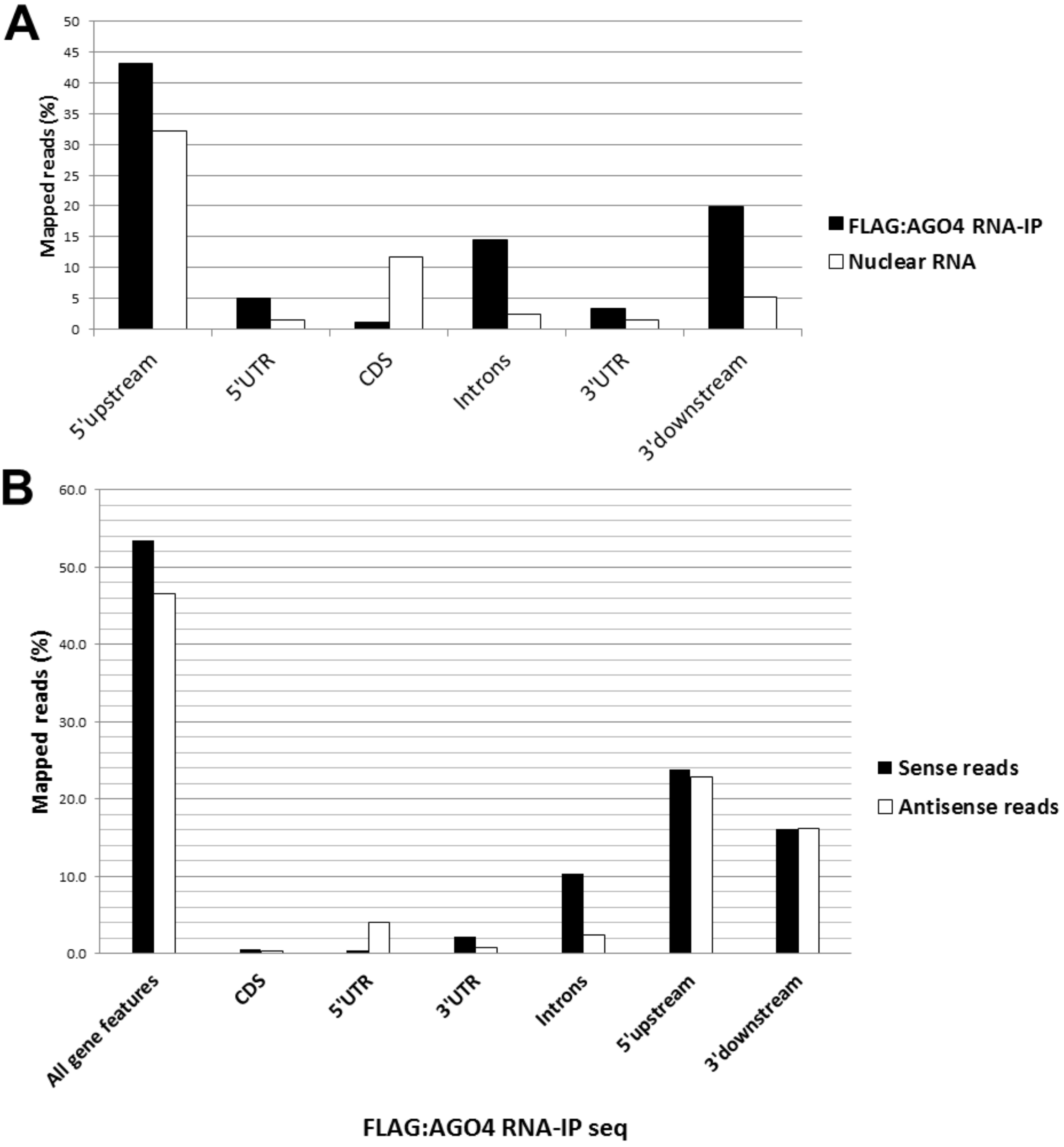

Figure S4: Read distribution across loci features between FLAG:AGO4 RNA-IP and nuclear RNA seq libraries. (A) Analysis of overall read distribution across loci features between FLAG:AGO4 RNA-IP and nuclear RNA seq library. Higher enrichment in coding sequence (CDS) reads was observed in nuclear RNA seq library. (B) Analysis of overall sense and antisense read distribution across loci features in FLAG:AGO4 RNA-IP library. UTR, untranslated region.

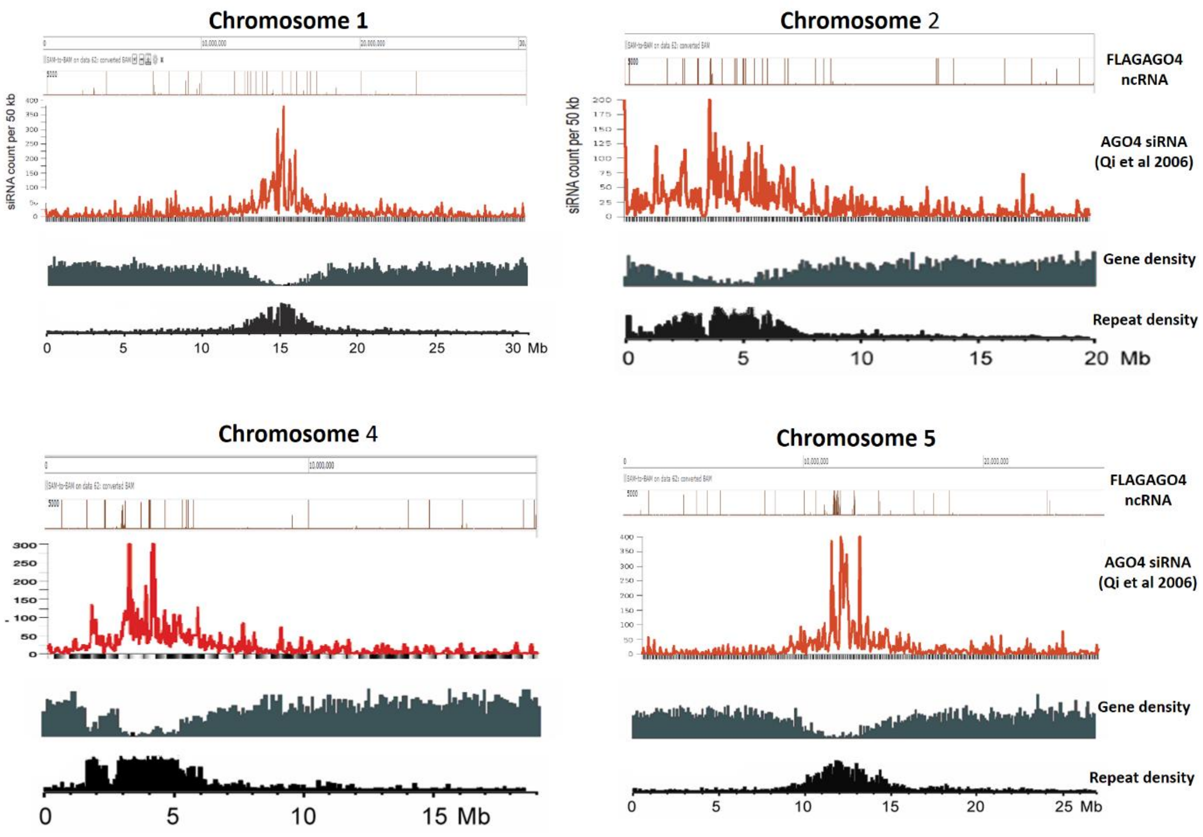

Figure S5: Chromosome wide density analysis of FLAG:AGO4 RNA-IP seq reads on Chromosome 1, 2, 4 and 5. Each peak represents a region on the Chromosome with at least 5000 reads. A clear overlap in peaks between FLAG:AGO4 RNA-IP seq reads (> 30 nt) and AGO4-associated 24 nt siRNAs data (Qi et al. 2006) [15] is observed, particularly in regions that are high in repeats but low in genes.

A psaA protein gene sequence

Original

ATGATGTTGTTAGAATTTTATATAGGTTTTTATGGTTTTGGTTTGTAAATGGA
TTTTTATGAGTTTTTAAAATATTTTTTATATTATGATTAATATTTTAGTTGGT
TTTATATATATGATTTGTGATTAGAAAAAGAATTGTGATATGTAAATGATG

C to T converted

ATGATGTTGTTAGAATTTTATATAGGTTTTTATGGTTTTGGTTTGTAAATGGA
TTTTTATGAGTTT**TTAA**AATATTTTTTATATTATGA**TTAA**TATTTTAGTTGGT
TTTATATATATGATTTGTGATTAGAAAAAGAATTGTGATATGTAAATGATG

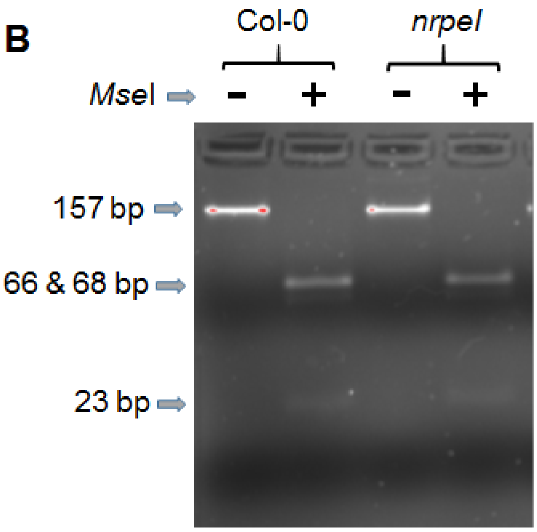

Figure S6: *Mse*I digestion confirms efficient bisulfite conversion of cytosines in the Col-0 and *nrpe1* samples used for bisulfite sequencing methylation analysis in Figure 4. (A) The chloroplast psaA gene sequence used for the analysis, which should be completely unmethylated due to the absence of DNA methylation in the chloroplast genome. Top, the original sequence; bottom, the sequence in which all cytosines are converted to uracil (appearing as T in PCR product), creating two *Mse*I restriction sites (TTAA). (B) The 157 bp PCR product was completely digested by *Mse*I to the shorter DNA fragments, indicating efficient bisulfite conversion. "-" and "+" indicate absence or presence of the *Mse*I enzyme in the samples. The 5% gel was prepared by using a mixture of 2.5% of low-melting agarose and 2.5% normal agarose.

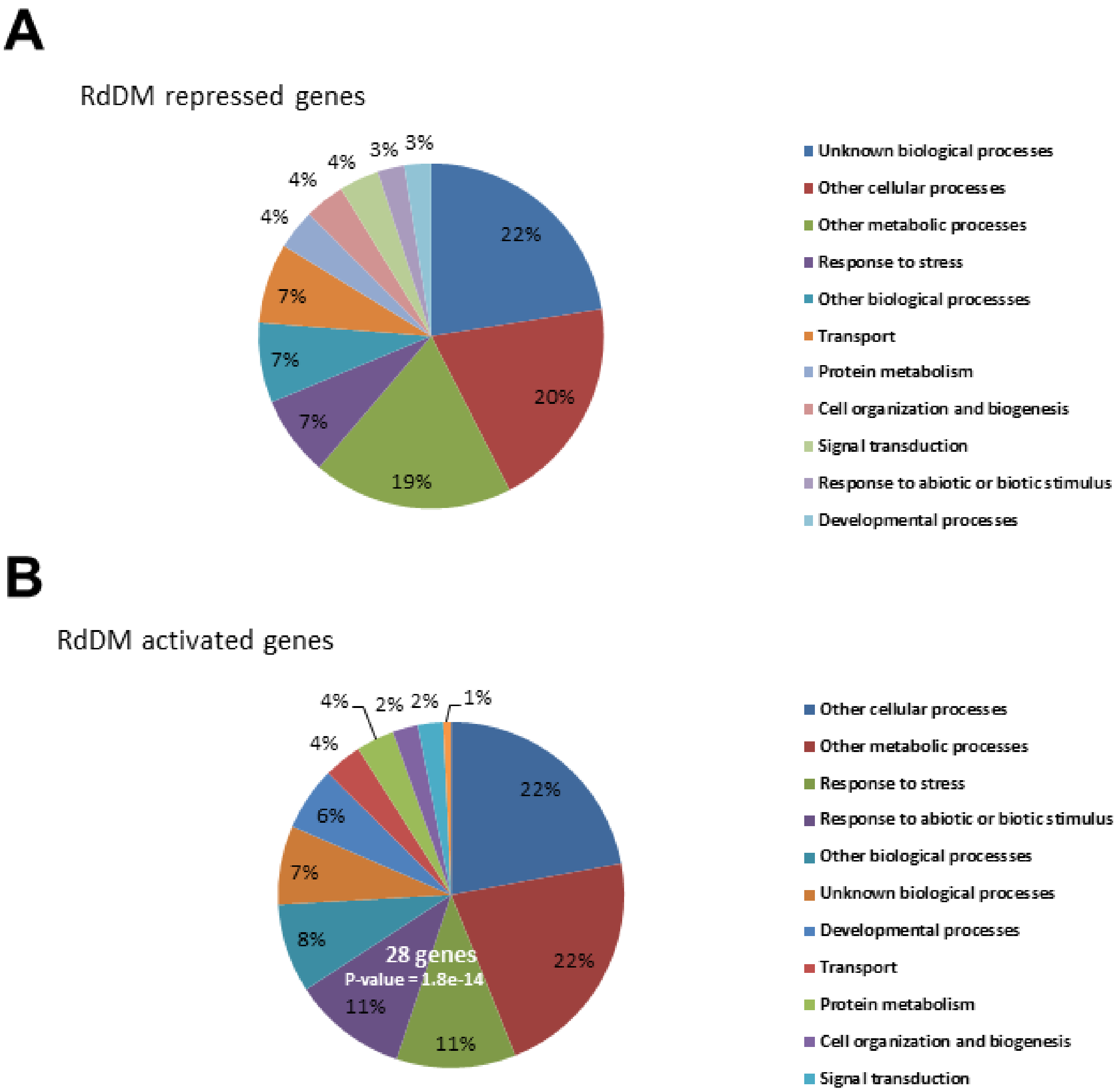

Figure S7: Go annotation analysis of RdDM repressed (A) and activated (B) genes according to biological function. Significant over-representation of response to stress and abiotic/biotic stimulus genes were found in RdDM activated genes. P-value was derived by performing the same analysis in agriGO.

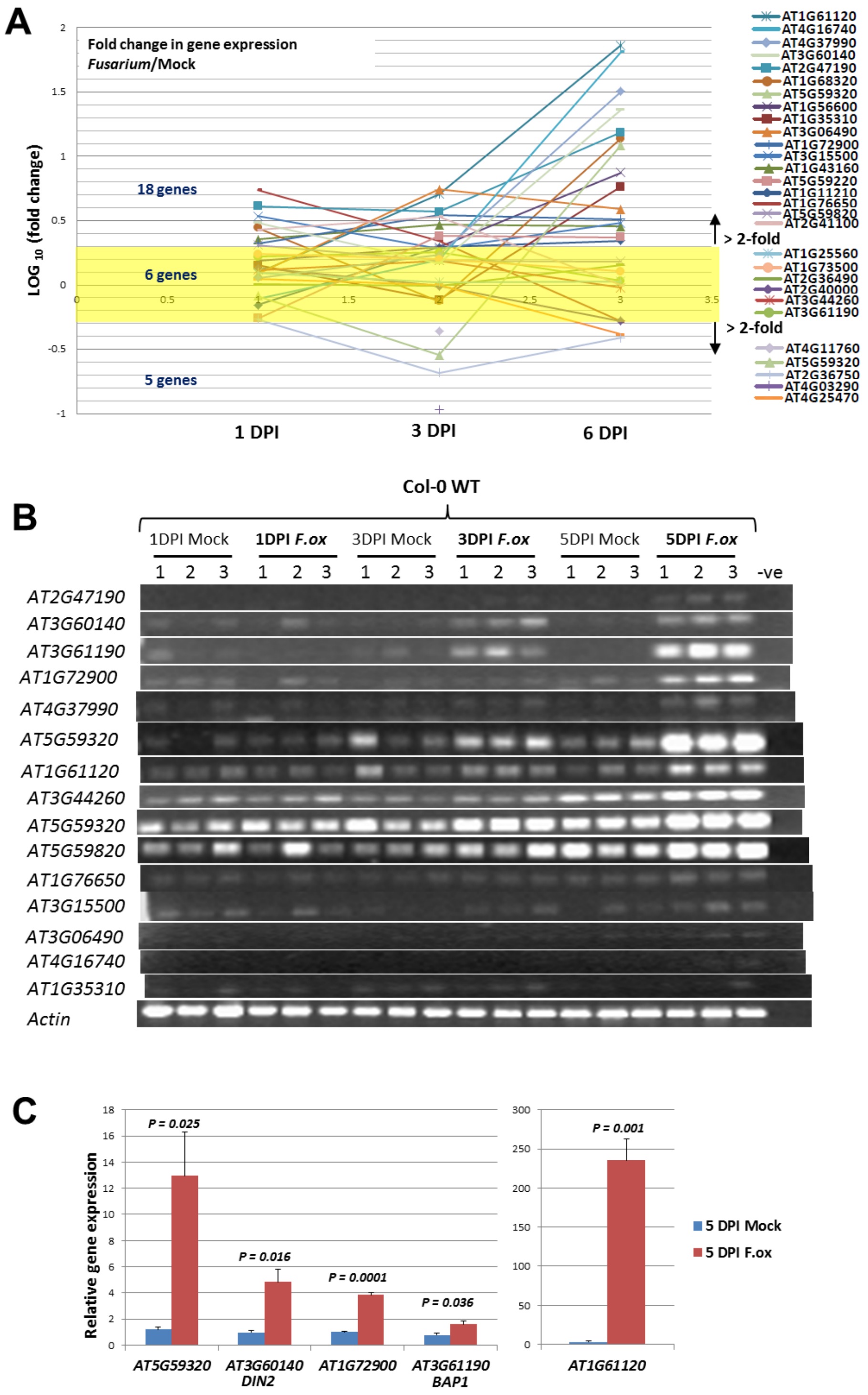

Figure S8: Expression analyses of putative RdDM-regulated stress response genes upon infection by *Fusarium oxysporum*. (A) Expression data in RPKM (Reads Per Kilobase Million) value for the 28 putative RdDM regulated stress response genes were extracted from an RNA seq dataset generated from Col-0 plants Fusarium/Mock infected at 1, 3 and 6 DPI (days post infection). Fold-change (Log_{10}) in gene expression in *Fusarium* infected Col-0 over Mock infected is shown for each gene. *AT5G59320* is represented in both up- and down-regulated categories as it is down by at least 2-fold at 3DPI but induced by over 2-fold at 6 DPI. (B) Semi-quantitative RT-PCR validation of 15 *Fusarium* induced genes identified from (A). (C) Validation of induction by real-time RT-qPCR of five *Fusarium* induced genes. Student's t-test detected significant differences at the P< 0.05 level.

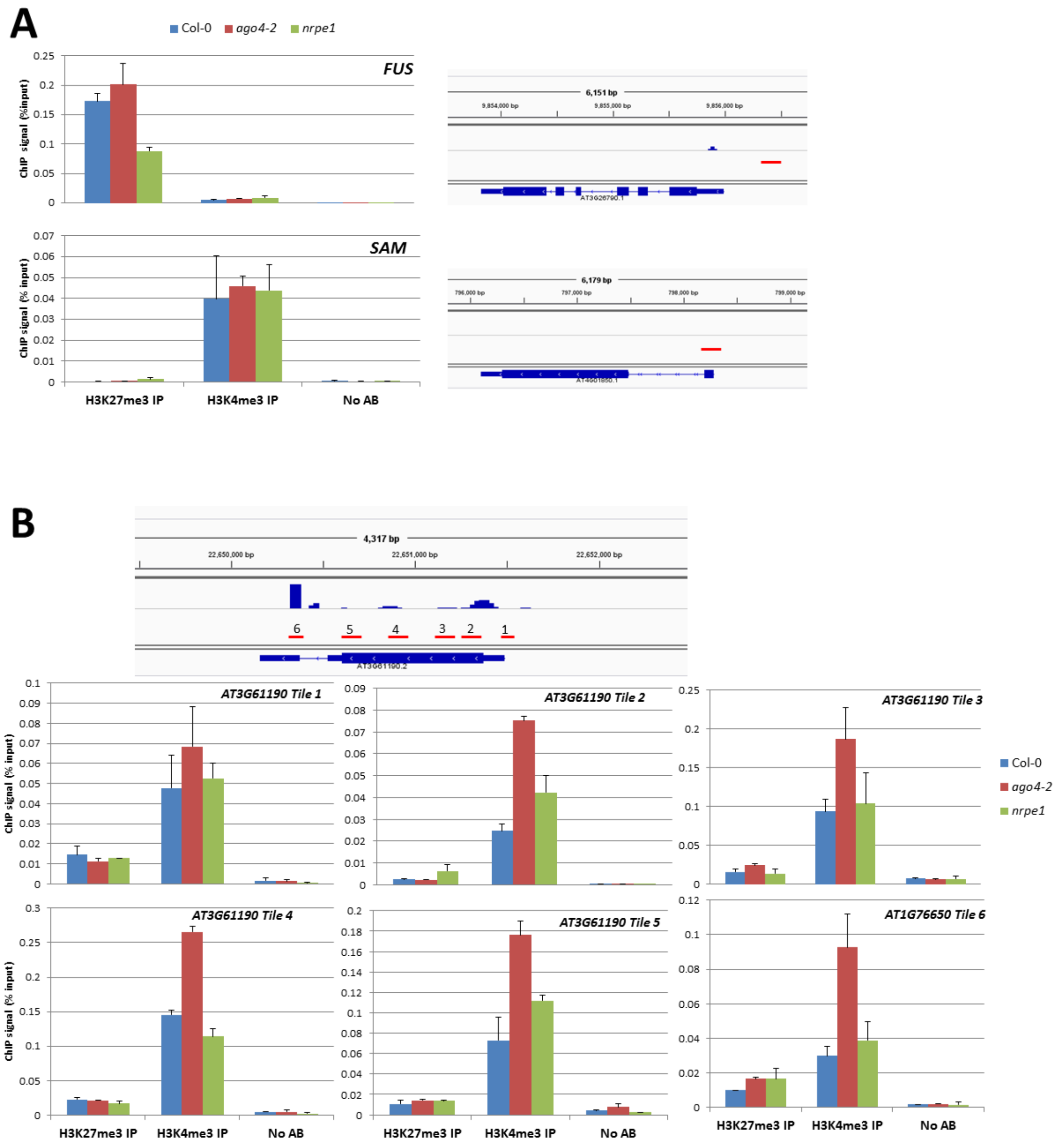

Figure S9: H3K27me3 and H3K4me3 ChIP assay. Histone H3 Lys 27 and Lys 4 trimethylation signal at (A) control genes *FUS3* (*AT3G26790*) and *SAM* (S-adenosylmethionine synthetase 2) (*AT4G01850*), and at gene body tiling regions of (B) *AT3G61190*. ChIP signals are normalized to the input signals. Error bars are the standard error of the mean from two biological replicates.

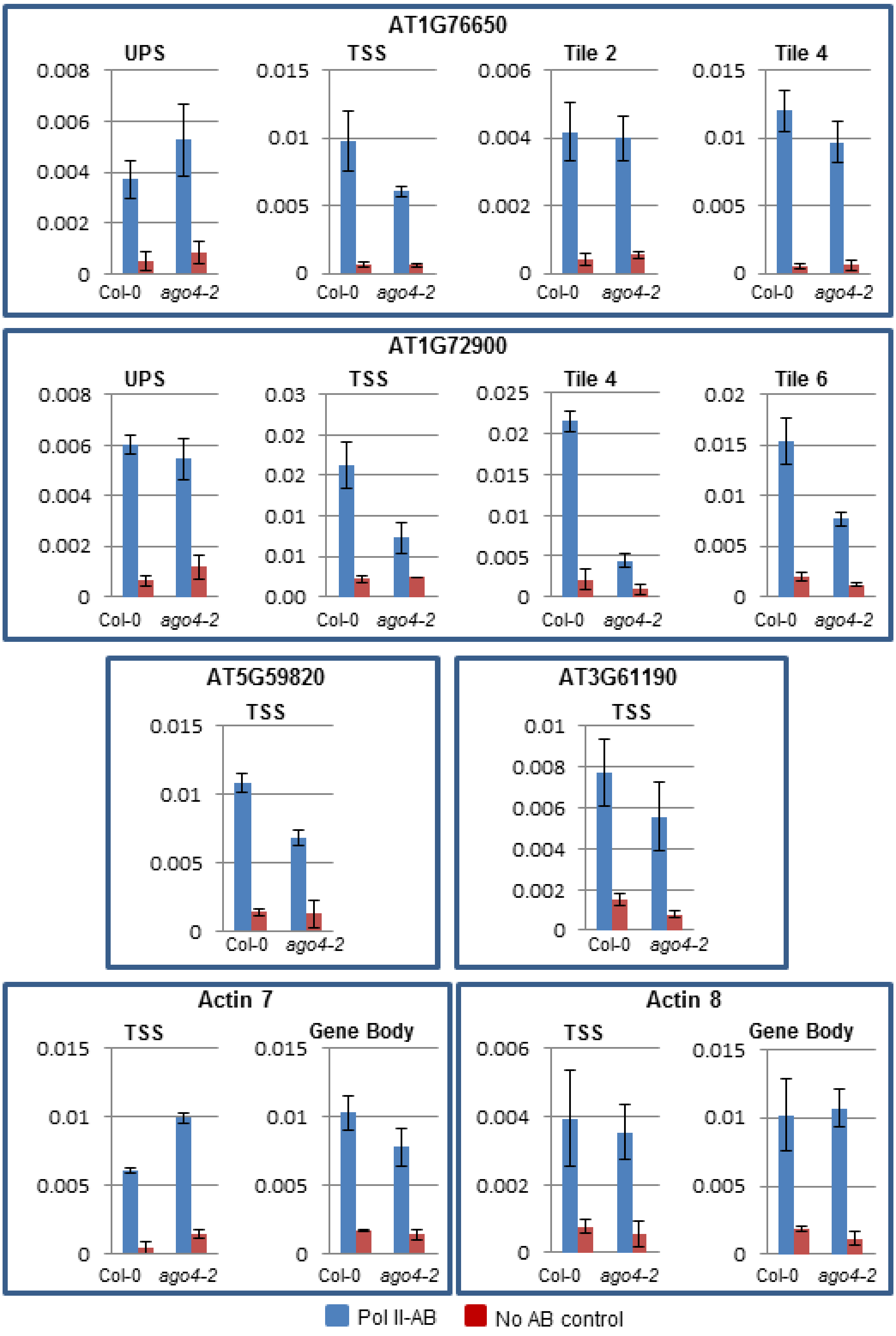

Figure S10: Pol II ChIP assay shows reduced Pol II occupancy in the transcription start site (TSS) of RdDM-activated genes in the AGO4 (*ago4-2*) mutant. The Actin 7 (AT5G09810) and Actin 8 (AT1G49240) genes are used as control. UPS, sequence 500 bp upstream of TSS.

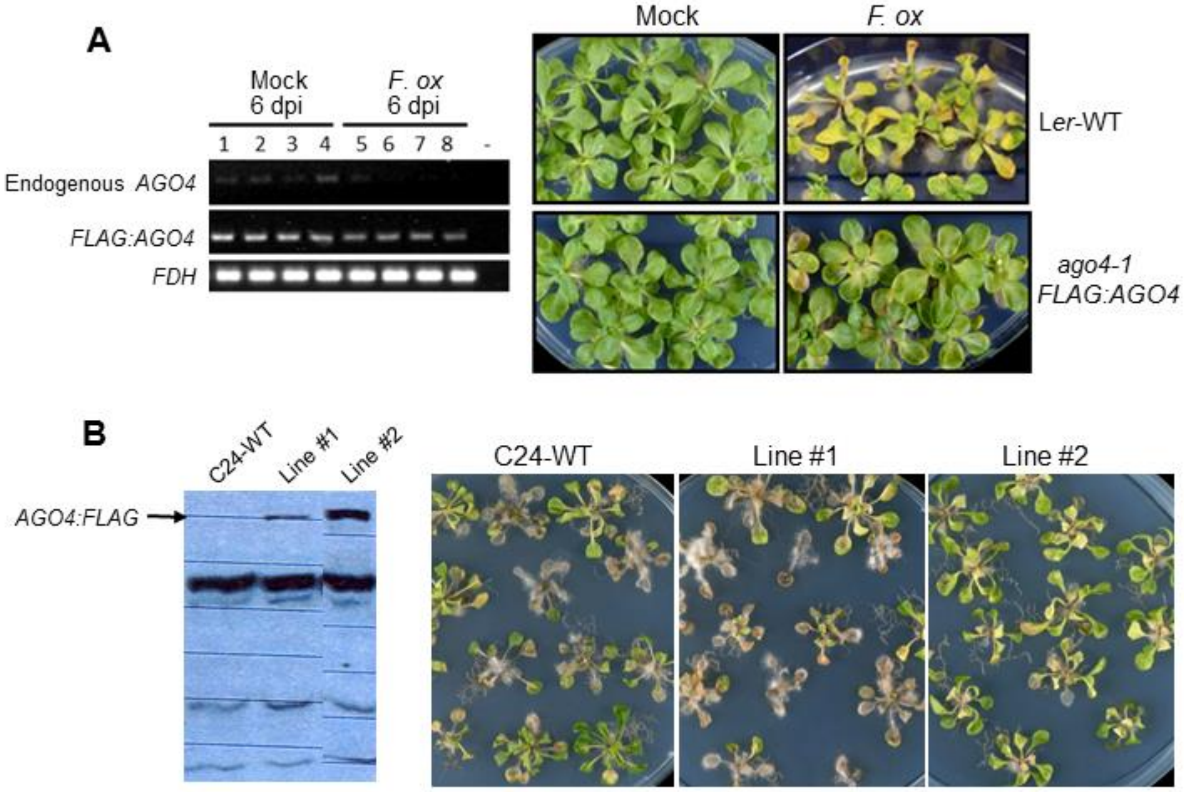

Figure S11: Transgenic *Arabidopsis* plants over-expressing AGO4 show enhanced resistance to *Fusarium oxysporum (F. ox)*. (A) Semi-quantitative RT-PCR of endogenous *AGO4* and transgenic *FLAG:AGO4* expression in *ago4-1 FLAG:AGO4* transgenic plants (left) and disease phenotype of representative wild type (L*er*) and *ago4-1 FLAG:AGO4* plants at 9 days post inoculation. *FDH* (*Formate dehydrogenase*) expression shown is the loading control. (B) Western blot analysis of AGO4:FLAG protein in C24 *AGO4:FLAG* transgenic plants (left; generated and kindly provided by Chris Helliwell), and disease phenotype of wild-type C24 and *AGO4:FLAG* transgenic plants at 10 days post inoculation (right). Note that Line #2 has a higher level of AGO4:FLAG protein than Line #1, and correspondingly, it shows enhanced *F. oxysporum* resistance.
